# Mass Spectrometry–Based Proteogenomics: New Therapeutic Opportunities for Precision Medicine

**DOI:** 10.1146/annurev-pharmtox-022723-113921

**Published:** 2023-09-22

**Authors:** Sunil K. Joshi, Paul Piehowski, Tao Liu, Sara J.C. Gosline, Jason E. McDermott, Brian J. Druker, Elie Traer, Jeffrey W. Tyner, Anupriya Agarwal, Cristina E. Tognon, Karin D. Rodland

**Affiliations:** 1 Knight Cancer Institute, Oregon Health & Science University, Portland, Oregon, USA; 2 Division of Hematology and Medical Oncology, Department of Medicine, Oregon Health & Science University, Portland, Oregon, USA; 3 Department of Medicine, Stanford University School of Medicine, Stanford, California, USA; 4 Pacific Northwest National Laboratory, Richland, Washington, USA; 5 Department of Molecular Microbiology and Immunology, Oregon Health & Science University, Portland, Oregon, USA

**Keywords:** oncoproteomics, phosphoproteomics, drug resistance, single-cell proteomics, proteogenomics

## Abstract

Proteogenomics refers to the integration of comprehensive genomic, transcriptomic, and proteomic measurements from the same samples with the goal of fully understanding the regulatory processes converting genotypes to phenotypes, often with an emphasis on gaining a deeper understanding of disease processes. Although specific genetic mutations have long been known to drive the development of multiple cancers, gene mutations alone do not always predict prognosis or response to targeted therapy. The benefit of proteogenomics research is that information obtained from proteins and their corresponding pathways provides insight into therapeutic targets that can complement genomic information by providing an additional dimension regarding the underlying mechanisms and pathophysiology of tumors. This review describes the novel insights into tumor biology and drug resistance derived from proteogenomic analysis while highlighting the clinical potential of proteogenomic observations and advances in technique and analysis tools.

## INTRODUCTION

As the molecular genetics revolution of the 1990s brought us a more detailed and mechanistic view underpinning malignancies, the hope of moving from empiric therapies to more targeted approaches by focusing on specific driver mutations began to flourish and motivate multiple clinical trials. While early results in the 1990s were mostly disappointing, the emergence of imatinib as a successful targeted therapy for chronic myeloid leukemia highlighted the importance of identifying the appropriate clinical subpopulation for precision medicine (1, 2). It could be argued that the success of imatinib in early clinical trials hinged on the availability of an easily detected molecular marker, the Philadelphia chromosome, for the identification of patients with the signature *BCR-ABL1* fusion (1). In a similar fashion, the selection of *HER2*-amplified breast cancer patients for trastuzumab therapy and estrogen receptor (ER)-positive patients for tamoxifen therapy represented an extension of the concept to solid tumors (3). In the two decades since these foundational applications, the use of genomics and/or transcriptomics to select the most appropriate targeted therapy has ushered in the era of precision oncology.

Early attempts at implementing precision oncology were bolstered by the efforts of The Cancer Genome Atlas (TCGA) and other large consortia focused on cataloging somatic mutations, copy number variations (CNAs), DNA methylation, and comprehensive transcriptomic analyses of specific tumor types (4). Transcriptomic data were mined to produce prognostic signatures associated with outcome as a step toward customizing the intensity of therapeutic interventions to the individual’s likelihood of progression (5). Early examples include the use of MammaPrint and OncoType DX to stratify breast cancer patients for aggressive therapy. More recently, these nucleic acid–based analyses have been extended to include comprehensive analyses of proteins and posttranslational modifications (PTMs) of tumors to create the new field of proteogenomics, which integrates protein-level measurements alongside genomics and transcriptomics, represented effectively by the Clinical Proteomic Tumor Analysis Consortium (CPTAC) and the International Cancer Proteogenome Consortium (ICPC). Beginning in the 2010s, CPTAC investigators provided in-depth proteogenomic characterization of selected tumor types that had been either previously analyzed by TCGA or prospectively collected for proteomic analyses (Table 1). These proteogenomic studies have enabled the tracking of information flow within tumors, including the identification of pathway-level changes associated with clinical outcomes. In this review, we summarize the results of these comprehensive proteogenomic analyses, provide new insights resulting from these studies, and highlight their existing and future clinical applications. We end with a discussion of current challenges limiting the translational benefits of proteogenomics and the technological advancements under development to address those challenges.

## THE PROTEOGENOMIC LANDSCAPE OF SOLID AND LIQUID TUMORS

### Solid Tumors

**Figure 1:**
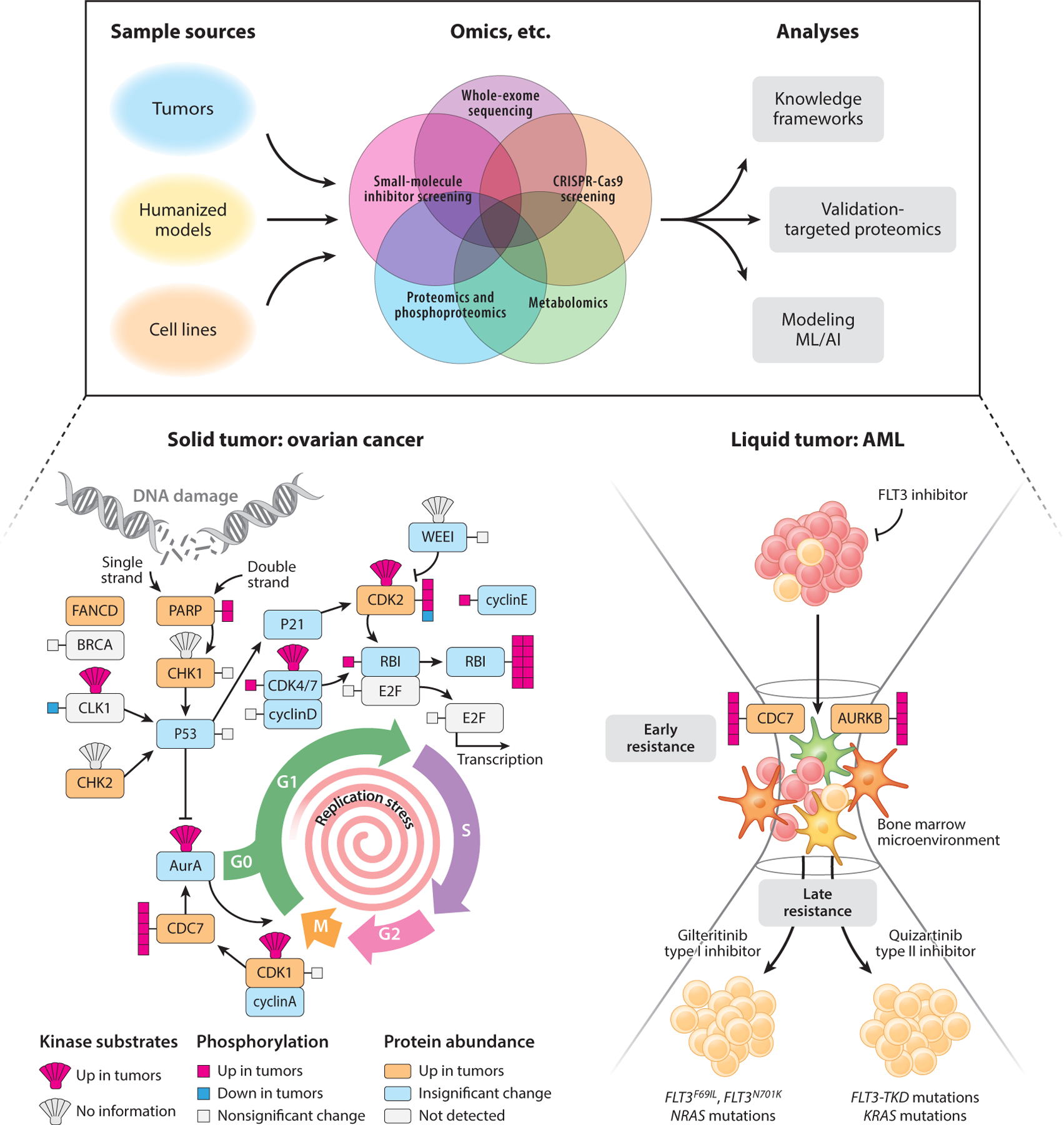
Chalk talk highlighting the multiomics approach our group has taken to reveal the underlying biology of solid and liquid tumors. New mechanistic insights and therapeutic targets have emerged from our global and phosphoproteomic profiling of ovarian carcinoma (12) and AML (37). Abbreviation: AML, acute myeloid leukemia.

Research conducted by CPTAC and ICPC investigators has enabled deep proteogenomic characterization of a series of solid tumors, narrowing the gap between cancer genotype and phenotype (6). Within this section we present examples, from an ever-growing literature of over 200 publications, that demonstrate the additional dimensions of cancer biology revealed by proteomic and phosphoproteomic analysis of solid and liquid tumors (Figure 1, Table 1). Novel insights from proteomics and phosphoproteomics that have potential therapeutic implications are highlighted. Studies focused on mapping the proteogenomic landscape of colorectal (7), breast (8), and ovarian (9) cancers were among the early efforts that enabled integration and aggregation of proteomic and phosphoproteomic analyses with corresponding genomic and transcriptomic data sets.

Zhang et al. (7) performed proteomics on 95 colorectal cancer (CRC) patient samples that were previously annotated through the TCGA. Integration of CNAs with transcriptomic and proteomic analyses uncovered many hot spots with potential driver alterations that could drive pheno-typic perturbations. Among these, the 20q amplicon was associated with the largest global changes at the messenger RNA (mRNA) and protein levels, revealing key driver genes such as *HNF4A*, *SRC*, and *TOMM34* that were previously underappreciated in CRC pathogenesis (7). Proteogenomic profiling of 110 prospective CRC samples led to the discovery of additional biomarkers, Rb phosphorylation, and dependence on glycolysis, which promote tumorigenesis (10).

Proteogenomic characterization of 77 patients with breast cancer provided a more complete picture of how previously TCGA-annotated CNAs (5q) and mutations (*TP53*, *PIK3CA*) manifest at the protein level (8). In addition to ERBB2, phosphoproteomics identified other highly phosphorylated kinases—cyclin-dependent kinase (CDK)12, PAK1, PTK2, RIPK2, and TLK2—that also contribute to the luminal tumor phenotype. Proteomics captured key protein pathways to distinguish breast cancer subtypes (particularly the stromal subtype) that are not reflected at the mRNA level (8). A more recent study integrated multiomics data from 122 treatment-naïve primary breast cancer samples (11). Deconvolution of mRNA and proteomic signatures indicated that a subset of luminal breast cancers had an overexpression of immune checkpoint and *STAT1*/*IFNG* genes, suggesting that there is potential for the use of immunotherapy within this setting. The authors also showed that Rb protein status correlated with response to CDK4/6 inhibition (11). These studies demonstrate that measuring the proteome is essential in overcoming the bottleneck that limits the translation of genomics to therapeutic strategies.

Proteomic characterization of 174 ovarian high-grade serous carcinoma (HGSC) patient samples, which had paired TCGA genomic analyses, demonstrated that proteomic utility is maximized when combined with genomics (9). One of the hallmarks of HGSC is chromosomal instability, as revealed by extensive CNAs. Proteins enriched in cell motility, invasion, and immune regulation were associated with CNAs and could be used to predict and stratify overall patient survival (9). A subsequent study from our laboratory extended proteogenomic analysis on 83 patient samples, implicating the activation of mitotic kinases and replicative stress as markers of ovarian HGSC (12).

Proteogenomic profiling of 103 clear cell renal cell carcinoma patient samples revised the current tumor classification to include immune-based subtyping, information that could not be gleaned from transcriptomics alone (13). Upregulation of proteomic signatures associated with hypoxia, glycolysis, epithelial-mesenchymal transition (EMT), and inflammation was observed alongside a stark downregulation in oxidative phosphorylation.

CPTAC studied 95 endometrial carcinomas at genomic, transcriptomic, and proteomic levels and found distinct protein signatures associated with histologic subtypes (14). They found a novel regulation of EMT by QK1, circular RNA, and ESRP2, which was associated with progressive disease. Although higher tumor mutation burden (TMB) is associated with better response in many tumors, their analysis found low levels of antigen-processing machinery (APM) in some TMB-high tumors, potentially limiting the efficacy of immunotherapy and suggesting that APM should be considered in future clinical trials.

Recent advances with targeted inhibitors and immunotherapy have begun to improve survival for lung adenocarcinoma (LUAD). CPTAC investigators studied 110 paired LUAD tumors with normal adjacent tissue using multiomics (15). Phosphoproteomics identified targetable kinases for combination therapy: SOS1 in *KRAS*-mutant LUAD and PTPN11 in *ALK-* and *EGFR*-mutant LUAD, the latter of which is being tested in clinical trials. Immunotherapy markers were explored as well, and an association of *STK11* mutations with immune-cold tumors was noted. Unlike LUAD, lung squamous cell carcinoma (LSCC) has not benefitted from targeted therapy and remains difficult to treat. Proteogenomic characterization of 108 LSCC samples revealed unique subtypes associated with EMT and phosphorylation signatures (16). Similar to CRC (10) and breast cancer (11), Rb protein amount and phosphorylation were suggested as markers for response to CDK4/6 inhibitors based upon proteomic data, and immune profiling revealed a spectrum of immune-cold to immune-hot tumors to potentially guide immunotherapy.

Glioblastoma multiforme (GBM) is the most aggressive brain malignancy and has a very high mortality. Ninety-nine treatment-naïve GBM tumors were analyzed by proteogenomics, metabolomics, and single-nuclei RNA sequencing (17). Phosphoproteomics identified increased activity of receptor tyrosine kinases (RTKs), protein tyrosine phosphatase non-receptor type 1 (PTPN1), and phospholipase C gamma 1 (PLCG1) signaling hubs, suggesting a potential therapeutic option. GBM could also be characterized into different immune subtypes, which could potentially be used in selecting immunotherapy. In contrast to GBM, pediatric brain tumors are both more rare and diverse. A proteogenomic analysis of 218 pediatric brain tumors was used to identify unique and common features for this rare disease (18). In particular, proteomics and phosphoproteomics were able to identify striking similarities between subgroups of craniopharyngioma and low-grade glioma tumors with *BRAF* V600E mutations, highlighting a potential therapeutic approach.

Pancreatic ductal carcinoma (PDAC) is a lethal cancer and difficult to treat, due to both the difficulty in resecting the pancreas and the lack of response to chemotherapy. Multiomic analysis of 140 PDAC samples and corresponding healthy tissue samples identified distinct glycoprotein expression associated with some *KRAS* mutations but not others, revealing a complexity beyond the simple presence of *KRAS* mutations. There was also an association of reduced endothelial cells, increased vascular endothelial growth factor (VEGF), and hypoxia-inducible factor (HIF) in immune-cold PDAC tumors.

Head and neck squamous cell carcinoma (HNSCC) can be broadly classified into human papilloma virus (HPV) associated and HPV negative, with the latter having a much worse prognosis. Multiomic approaches were used to characterize 108 HPV-negative HNSCC tumors and matched normal adjacent tissue samples (19). Epidermal growth factor receptor (EGFR) is a known target in HNSCC, but amplification does not always predict response. Huang et al. (19) found that overexpression of EGFR ligands as measured by proteomics was more predictive of both EGFR activity and clinical response to EGFR antibodies such as cetuximab, which block ligand binding. With respect to immunotherapy, HPV-negative HNSCC had low levels of antigen presentation, leading to immune-cold tumors. Taken together, proteomic analyses have identified novel therapeutic targets, informed downstream validation experiments, and provided a detailed landscape for solid tumors.

### Liquid Tumors

Acute myeloid leukemia (AML) is difficult to treat and has an overall five-year survival rate of less than 25% (20). Cytotoxic chemotherapy has remained the primary treatment for decades, with minimal improvement in patient outcomes. Recent attempts to create small-molecule kinase inhibitors, similar to imatinib (1, 21), have met with limited success, owing to the heterogeneity of AML (22, 23).

A number of studies have evaluated the genomic landscape of AML with respect to mutational and drug response profiling (22, 24–26). However, the underlying biology connecting genomic aberrations with drug response is not easily apparent; proteomic analyses have helped bridge this gap (27). Proteomic profiling has identified a novel Mito-AML (28) and age-dependent alterations contributing to chemoresistance (29). Posttranscriptionally regulated proteins have been identified in genetically defined subsets (e.g., KDM4 isoforms in IDH1/2 mutation–positive patients or elevated nuclear importins in NPM1-mutated AML patients) (30). These examples underscore the ability of proteomic data to provide novel mechanistic insights.

Kinase-substrate enrichment analysis (KSEA), which evaluates mass spectrometry (MS)-based phosphoproteomic data and infers kinase and associated network activity, was originally developed using AML models (31). KSEA identified phosphatidyl inositol 3 kinase (PI3K), casein kinases, CDKs, and p21-activated kinases as the kinases that are most frequently enriched in AML. Similarly, clustering of differentiation marker expression has been used to infer remodeled AML kinase-signaling networks during differentiation, identifying increased activity of prosurvival pathways regulated by MAP2K1 and protein kinase C (PKC) (32). Based on a study of primary patient samples, a phospho-signature containing seven validated peptides was found to predict FLT3 inhibitor response in patients with 78% accuracy (33). Selected reaction monitoring (SRM) or immunological detection assays of peptide signatures would enable clinical testing of biopsies. In the future, predictive phospho-signatures could facilitate personalized drug selection or real-time monitoring to enable early detection of drug resistance.

MS-based proteomic approaches mapped the effects of targeted agents on AML cell lines and primary samples and identified key pathways affected by drug treatment (33–35) and mutational status (36) or targetable pathways associated with drug resistance (37, 38). We previously used computational approaches to integrate proteomic profiling with genomics, transcriptomics, and small-molecule inhibitor sensitivity data sets to create models that recapitulate patient biology, which can be leveraged to prioritize treatment strategies (37, 39).

Pluripotent self-renewing leukemic stem cells (LSCs) must be fully characterized to develop therapies that eradicate residual disease and achieve long-term remissions. MS analyses have revealed LSC-specific changes in oxidative phosphorylation, adhesion molecule composition, and RNA processing properties (40). Similar findings were uncovered from in-depth proteomic studies performed on 47 adults and 22 pediatric AML samples taken throughout disease progression (41). Mitochondrial ribosomal protein and subunits of the respiratory chain complex were enriched at relapse, suggesting a role for altered energy metabolism.

Proteogenomic analyses on ETV6/RUNX1-positive, hyperdiploid pediatric acute lymphoblastic leukemia samples showed underexpression of CCCTC-binding factor (CTCF) and cohesins, master regulators of chromatin architecture, suggesting a potential mechanism for dysregulating gene expression (42).

To uncover nongenetic mechanisms of multiple myeloma lenalidomide resistance, global tandem mass tag (TMT)-based proteomic and phosphoproteomic analyses were performed on paired pretreatment and relapsed samples. A CDK6-governed resistance signature was uncovered, which included high-risk factors such as thyroid hormone receptor interactor 13 (TRIP13) and ribonucleotide reductase catalytic subunit M1 (RRM1) and identified synergy between CDK inhibition and lenalidomide treatment (43). Proteomic analyses performed on liquid tumors have uncovered numerous classification and response signatures with translational value. Proteomic-based tests could be leveraged in the future to identify targeted therapies for individual patients, to monitor drug resistance, and to detect disease recurrence.

## PROTEOMIC ANALYSIS OF BLOOD AND BODY FLUIDS

In clinical laboratory testing, blood or body fluids are most widely used to determine disease diagnosis and prognosis. One advantage of liquid malignancies is that blood samples can be easily collected in the clinic, providing viable cells for downstream proteomic applications. This is particularly useful for longitudinal analyses to understand response and resistance over the course of treatment. For example, liquid chromatography–tandem mass spectrometry (LC-MS/MS) was used to identify changes in global and phosphorylated proteins associated with AML relapse in 41 patients due to the availability of serial patient samples. This study demonstrated that relapse was associated with increased expression of RNA processing proteins and decreased expression of V-ATPase proteins. Further, there was an increase in phosphorylation events catalyzed by CDKs and casein kinase 2 (44, 45). Since these pathways can be targeted therapeutically, this may inform clinical approaches to identify and target resistance pathways.

The ability to isolate various cell types from blood samples using flow cytometry also enables proteomic profiling at a subpopulation level. In a recent study, proteomic characterization was performed to identify specific vulnerabilities of CD34^+^ LSCs that can be leveraged therapeutically (40). Similar proteomic analyses were performed on subsets of monocytes, identifying over 5,000 proteins potentially associated with disease processes (46). While the analysis of blood samples is relatively straightforward, analyzing bone marrow biopsies from leukemia patients is still necessary to understand the full tumor ecosystem, which encompasses communication between the leukemic cells and neighboring cells of the marrow microenvironment. We have used pre- and on-treatment AML bone marrow samples to identify early biomarkers that can be harnessed to circumvent the development of FLT3 drug resistance (37).

While proteomic applications have been readily incorporated into studies of drug resistance within liquid tumors, this application remains a largely uncharted territory in solid tumors, at least partially due to the invasive nature of collecting a single biopsy or longitudinal samples. Despite these challenges, MS-based proteomic strategies have been applied to profile many solid tumors as discussed above (47).

More recently, proteomic approaches have also been developed to analyze extracellular vesicles (EVs) and body fluids for the presence of disease progression and drug resistance markers. Proteomic profiling of EVs isolated from Ewing sarcoma cell lines compared to healthy human plasma led to the discovery of the Ewing sarcoma–specific markers CD99 and nerve growth factor receptor (NGFR) (48). Parallel analyses of EVs in breast cancer identified subtype-specific biological processes and molecular pathways, including hyperphosphorylated receptors, kinases, and defined protein signatures that closely reflect the associated clinical pathophysiology (49).

Examination of body fluids such as urine, saliva, tears (50), and plasma via proteomics has similarly yielded the identification of disease biomarkers and precision oncology approaches (47). Cerebrospinal fluid (CSF) surrounds the brain and spinal cord, providing mechanical and immunological protection; however, it is not sampled as often as blood in central nervous system malignancies as its collection is significantly more invasive. In a recent study, 251 CSF samples from patients with four types of brain malignancies and healthy individuals were analyzed by proteomic analysis. By integrating CSF data with proteomic analyses of corresponding tumor tissue and primary glioblastoma cells, CSF biomarkers such as chitinase-3-like protein 1 and glial fibrillary acidic protein were identified (51). Despite challenges in sample accessibility and collection, proteomic characterization of a plethora of body fluids has fueled the discovery of new biomarkers and therapeutic targets.

Novel methods in single-cell MS-based profiling (52) and subcellular or EV proteomic evaluation (53–56) are also uncovering new biology and pathways for therapeutic targeting. Computational workflows such as SCeptre (Single Cell proteomics readout of expression) have enabled the normalization of global single-cell MS data from up to 1,000 cells. These new single-cell techniques can be deployed on bulk or enriched populations of cells, enabling the exploration of leukemia cell heterogeneity (52). Proteomics performed on subcellular isolates from AML cells such as plasma membrane (54), the nucleus (53, 55), and EVs (56) have enabled leukemia subclone tracking by identifying 50 leukemia-enriched plasma membrane proteins (54), helped to identify novel therapeutic targets such as the nuclear protein S100A4, and supported the evaluation of drug combinations such as the combination of the nuclear export inhibitor selinexor with the MDM2 inhibitor nutlin-3a or the AKT inhibitor MK-2206 (53, 55).

Recently, several studies successfully harnessed the power of comparative spatial proteomics as a discovery tool to unravel disease mechanisms in solid tumors. While spatial proteomics approaches are difficult to implement in liquid tumors, this approach can be used for functional identification of rare tumors, tumor-infiltrating immune cells, and dissection of cellular mechanisms (57). As an example, integration of spatial proteomics with traditional phosphoproteomics uncovered that fibroblast growth factor receptor 2 beta (FGFR2b) stimulated by its ligand fibroblast growth factor 10 (FGF10) activates mammalian target of rapamycin (mTOR)-dependent signaling and ULK1 in recycling endosomes, resulting in suppression of autophagy and cell survival (58). Going forward, the improvement of single-cell proteomics along with spatial proteomics can significantly deepen our understanding of disease biology, particularly when combined with drug response and clinical outcome data.

## MASS SPECTROMETRY TECHNOLOGIES AND ADVANCES

**Figure 2:**
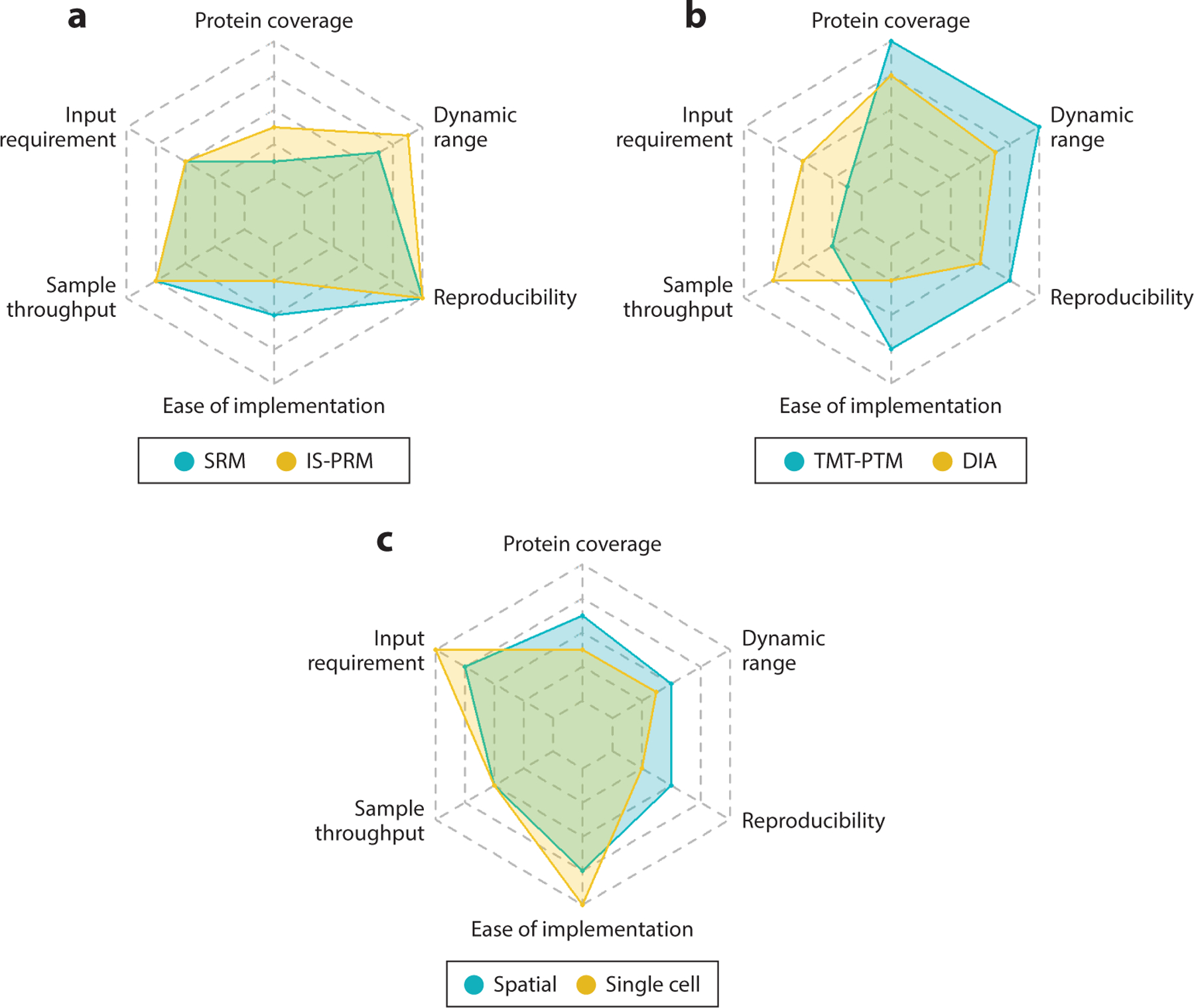
Radar plots comparing the analytical figures of merit for proteomics modes. (*a*) Targeted approaches include selected reaction monitoring (SRM) and internal standard triggered–parallel reaction monitoring (IS-PRM). (*b*) Discovery/global approaches include tandem mass tag with serial posttranslational modification enrichment (TMT-PTM) and data-independent acquisition (DIA). (*c*) Spatial and single-cell approaches. Protein coverage refers to the number of proteins that can be quantified in an experiment. Dynamic range is defined as the concentration range of proteins that can be accurately quantified. Reproducibility is the coefficient of variance of replicate analyses. Ease of implementation indicates how accessible the methodology is to general practitioners. Sample throughput is the number of patient samples that can be analyzed per unit time. Input requirement is defined as the amount of specimen needed for analysis with single-cell methods having the smallest sample requirement.

Large-scale proteomic tumor characterization typically utilizes LC-MS/MS-based platforms with offline fractionation/concatenation for comprehensive quantitative analysis of proteins and PTMs (7–19, 59–67). Isobaric labeling significantly improves analysis throughput by barcoding individual samples for multiplexed MS analysis, and by implementing a common reference strategy, it also allows samples in a large cohort to be effectively quantified (8, 9). The CPTAC and ICPC studies quickly improved from the earlier 4-plex iTRAQ (68) (isobaric tags for relative and absolute quantitation) analysis to 11-plex TMT (69) (tandem mass tag) or 16-plex TMT (70) analysis. Further improvements are expected based on the new 18-plex TMT (71) and other even higher multiplexing (72) methods. Besides isobaric labeling and data-dependent acquisition–based workflows, with significant recent technical advances, data-independent acquisition (DIA) (73) is also becoming a viable alternative for comprehensive, single-shot analysis of proteins and PTMs such as phosphorylation and glycosylation (61). A comparison of the most relevant analysis methods and their relative strengths and weaknesses is shown in Figure 2.

After the initial proof-of-concept proteogenomic studies (7, 62), simultaneous analysis of proteins and phosphorylation became the baseline to gain critical insights into signaling network activities in cancer. In addition to increasing throughput, isobaric labeling also enables integrated protein and PTM analysis (i.e., from exactly the same sample) while significantly reducing the sample input requirement for individual samples. The latter has important implications for the effective utilization of size-limited clinical samples because comprehensive analysis of PTMs, generally present at substoichiometric levels, requires enrichment from large amounts of sample. More PTMs can be added to a basic integrated workflow (74) where 5% of TMT-labeled peptides are used for analysis of unmodified peptides and 95% of peptides are subjected to phosphopeptide enrichment using immobilized metal affinity chromatography (IMAC). For example, the enrichment of ubiquityl peptides (75) and tyrosine-phosphorylated peptides (76) using antibody-based methods can be added before IMAC; acetyl peptides (11, 14, 16, 17) and glycosylated peptides (60) can be enriched from IMAC flow-through using antibody- and chromatography-based methods, respectively. Additionally, analysis of the immunopeptidome (77) may be added to the beginning of this integrated workflow for cancer types harboring significant mutations. Discoveries made on protein and PTM abundance changes can be confirmed in additional cohorts using targeted proteomics methods (78) such as SRM (79) and parallel reaction monitoring (80–82).

The proteomics workflows mentioned above enable deep protein and PTM analysis; however, they necessitate bulk sample processing, which precludes investigating the role of different cell populations and tumor heterogeneity in disease (83, 84). Recent technological improvements have enabled extension of MS-based proteomics to spatially resolved (85–93), cell type–resolved (91, 94, 95), and single-cell (52, 96–100) measurements. Cell types or regions of interest can be isolated using cell sorting (101–103) or laser capture microdissection (104, 105) and collected into microwell plates for further processing or coupled directly to recently developed chip-based platforms (106–108). The samples are then prepared with optimized protocols aimed at minimizing sample losses to surfaces and optimizing digestion kinetics at low sample concentrations, for example, nanoliter droplet processing (109, 110), advanced microfluidic devices (111), and microplate approaches (99, 112).

After preparation, samples can be analyzed by either label-free quantification (LFQ) or isobaric labeling quantification approaches. For isobaric labeling, employing a carrier approach, which involves leveraging a TMT channel with significantly higher loading of peptides with similar composition to the study samples, has greatly improved sensitivity (100, 113), albeit at the cost of deteriorated quantitation (114–116). LFQ provides more accurate quantification; however, without sample multiplexing, less material is available for analysis, which requires further workflow customization (98, 99, 117, 118). The maturation of DIA algorithms (119–121) and analysis pipelines has resulted in significant improvements in peptide identification efficiency and reduced missingness even when the signal is limited (111, 122). Another emerging technology that promises to significantly improve LFQ sensitivity is the integration of ion mobility separations between the LC and the MS (99, 123–125).

Currently, applications in the spatial and single-cell domains have been largely limited to global proteomics measurements, but efforts aimed at the miniaturization of PTM enrichment are well underway (126, 127). Perhaps even more exciting, advances in nanodroplet processing platforms when combined with ion mobility have produced the first demonstration of proteomics and transcriptomics from the same single cell, opening the possibility of single-cell proteogenomic measurements (128).

## COMPUTATIONAL DATA INTEGRATION AND BIOINFORMATIC CHALLENGES

The integration and interpretation of proteogenomic measurements, and ultimately, the test of their value, comes from the development of novel computational approaches. Data integration is first challenged by the fact that data exist on varying scales—genetic mutations are often assigned as discrete types of calls depending on the type of change in the DNA sequence (129, 130), while transcript measurements represent absolute changes in the mRNA relative to the length of the transcript and the depth of sequencing (131), and protein measurements are log ratio values representing the amount of sample measured relative to a standard control (132). Methods to overcome these challenges depend on the type of analysis at hand: Namely, nonnegative matrix factorization helps identify clusters of samples that behave similarly across scales (133–135) using all types of omic data, differential expression analyses can be performed between experimental conditions, and overlap between those features compared. Even after normalization, omic measurements do not agree as often as expected, as genetic mutations can fail to confer changes in expression (136, 137), changes in RNA expression may not result in actual protein changes (137, 138), and PTMs can be altered without changes in protein levels (139). As such, it is necessary to evaluate all omics measurements in an integrated fashion. These integrated approaches map omics measurements or changes to published data by mapping changes directly to the transcriptomic (140), proteomic (141), or phosphoproteomic networks (142, 143) or by comparing changes to lists of genes that represent pathways (144) or signatures of response. These approaches have enabled the study of proteogenomics in cancer to identify findings that are greater than the sum of their parts—an integrative and aggregative approach.

Despite these advancements in computational analysis tools, proteomics introduces a unique computational challenge to precision medicine. Most precision medicine–based approaches rely on large patient cohorts to identify mutated genes that signify a change in prognosis or treatment response. In gene expression studies, this approach has expanded beyond single genes to identify signatures or groups of transcripts that can be used to infer patient response (145, 146). In proteomics, however, identification of signatures or biomarkers is stymied by (*a*) the relative quantitative nature of MS, requiring shared reference samples (147, 148); (*b*) increased difficulty of detection for some proteins/peptides, resulting in potential biomarkers being missed (149); and (*c*) diversity in sample processing that causes large batch effects between downstream analyses. Experimental techniques such as sample pooling, common reference samples, and MS undersampling can lessen the impact of missingness in these data sets caused by absent peptides/proteins (150–152), but many bioinformatic challenges remain due to the numerous steps required for data processing and optimization.

**Figure 3:**
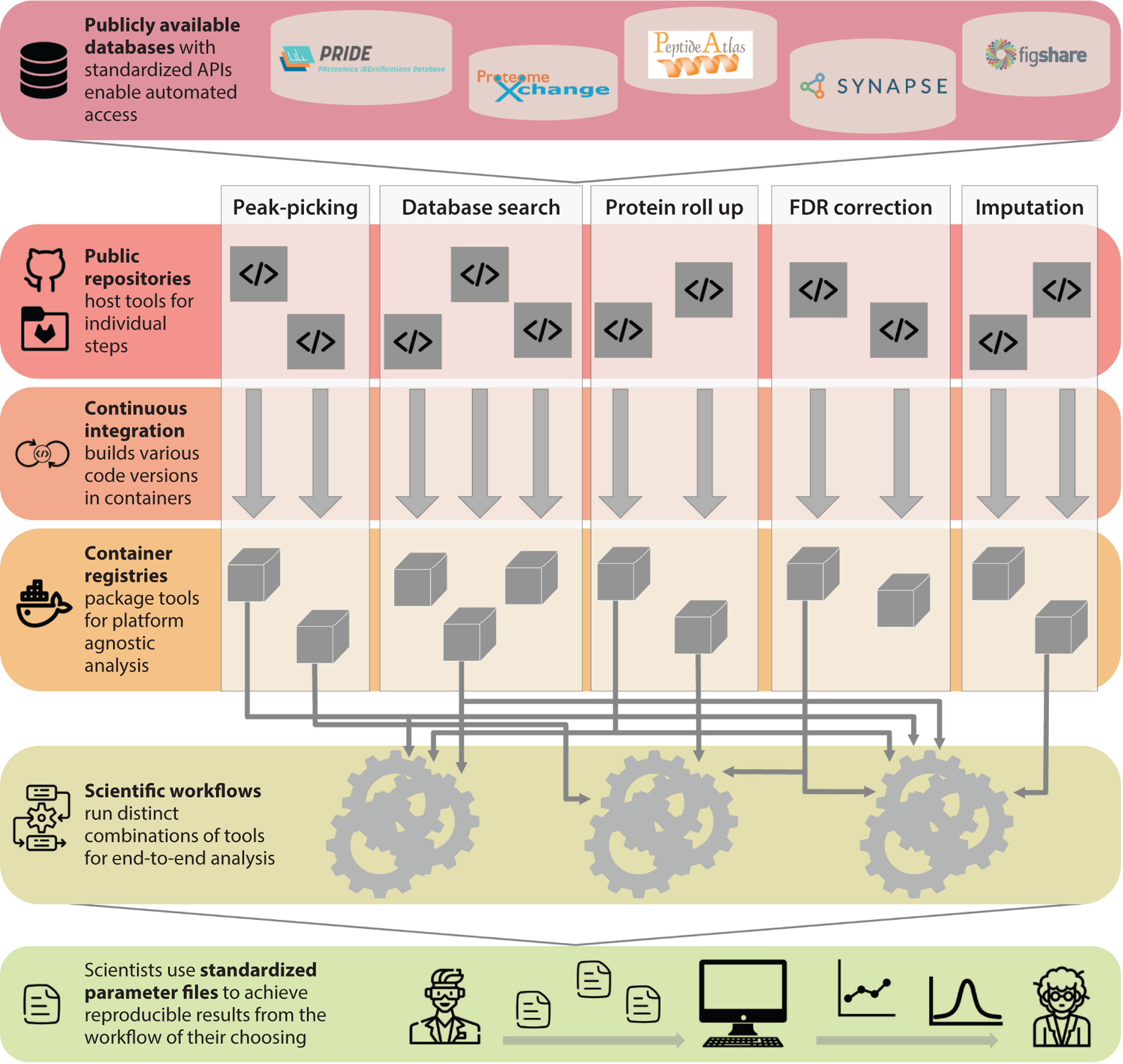
Summary of scientific workflow tools to enable precision medicine proteomic analyses. (*Top*) Standardized databases enable storage of data in machine-readable formats. (*Middle*) Public repositories, continuous integration, and container registries enable tool developers for each of the five steps (from left to right) of analysis to create state-of-the-art tools and also maintain all versions. (*Bottom*) Scientific workflow languages link tools in sequence as needed by scientists, who provide standardized parameter files to reproduce analysis uniformly across large cohorts. Abbreviations: API, Application Programming Interface; FDR, false discovery rate.

Batch effects in any high-throughput computational workflow stem from the numerous steps in the data analysis pipeline, each of which can be done by a handful of tools that each give different results. These steps, summarized in Figure 3, include (*a*) peak selection (153), (*b*) searching databases for peptide matches (154, 155), (*c*) mapping peptides to proteins, (*d*) filtering for false discovery, and (*e*) imputation of missing data (156–159). Since each step can be applied with different parameters, or with entirely different databases, the pooling of data across patient cohorts needed for precision medicine requires accurate accounting of the tools and data utilized.

Many tools have been developed to enable provenance across methodological variables. These tools break down into five categories, independently colored in Figure 3, and include (*a*) standardized data repositories with open application programming interfaces for data retrieval such as PRIDE (160), ProteomeXchange (161), Figshare (https://figshare.com/), and Synapse (https://www.synapse.org/); (*b*) open-source code repositories such as GitHub, GitLab, or Bit-bucket that enable sharing of methods; (*c*) continuous integration tools that automate container building and check for quality; (*d*) container registries that store versioned images to run the tools such as Docker Hub and BioContainers; and (*e*) scientific workflow repositories that enable storage of the precise steps that run the necessary tools in the necessary order using languages such as the common workflow language (162), Nextflow (163), and the workflow description language (163). With these tools in place, scientists need only the standardized parameter files and the workflow language/container tools installed on their machine to run analyses. Proteomics analysis frameworks have only scratched the surface of scientific workflow development (164, 165), but as these methods become more popular, larger cohorts can be harmonized for precision medicine analyses (166).

## CONCLUSIONS AND FUTURE DIRECTIONS

**Figure 4:**
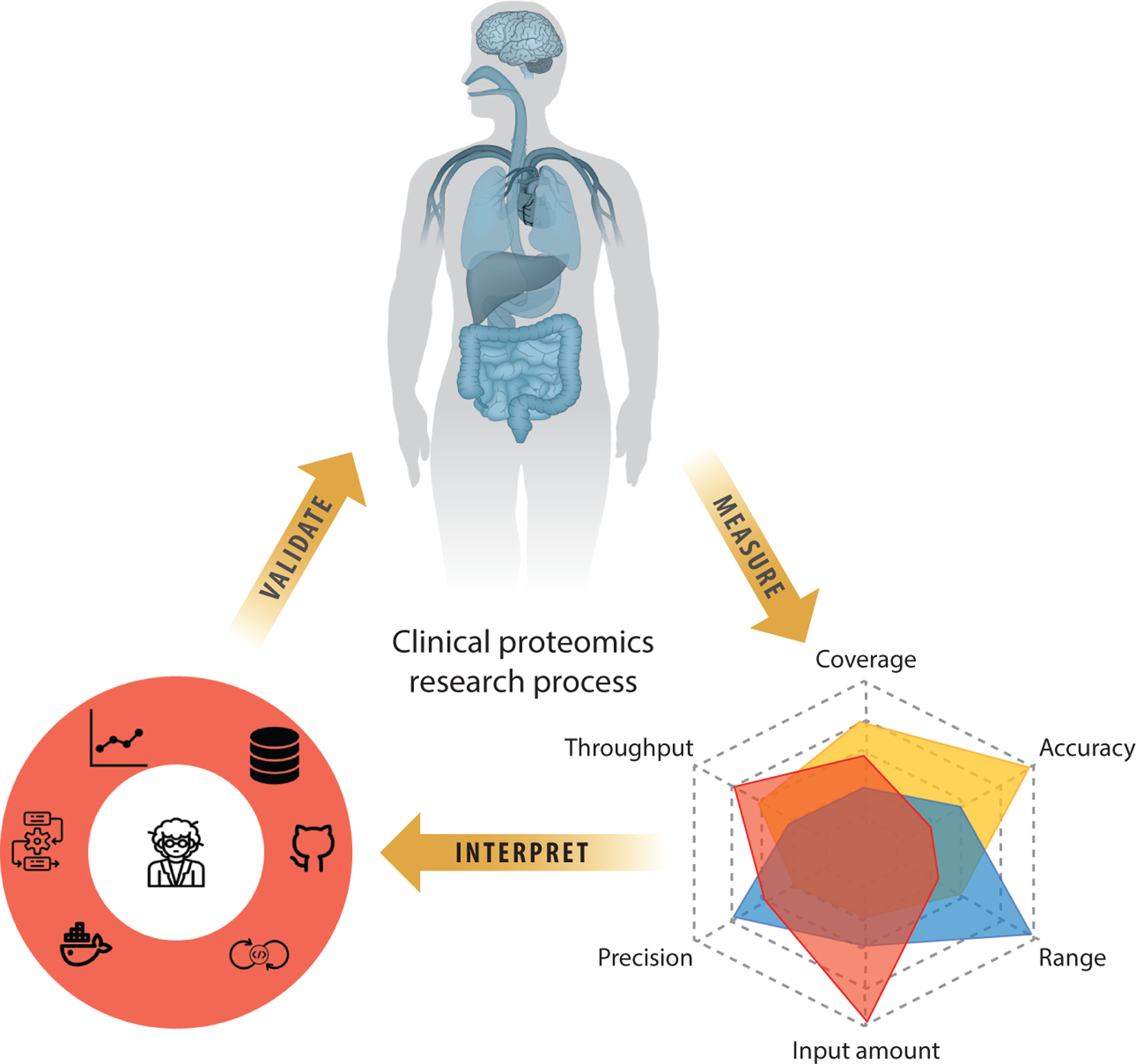
Leveraging proteogenomics in precision medicine. (*Top*) Sample procurement requires proper processing of clinical samples. (*Right*) Selection of mass spectrometry technology requires balancing trade-offs from each technology. (*Left*) Mapping proteogenomic measurements to clinical outcomes requires assembling diverse bioinformatic tools.

MS analyses of primary tumors have clearly been instrumental in major discoveries that have impacted our understanding of cancer biology, diagnostic precision, prognostication, and development of new therapeutic strategies (Figure 1, Table 1). Proteogenomics data have identified features of tumors that were undiscernible through genetic or transcriptomic analyses, and integration of all data types has led to additional discoveries, following the analytical process outlined in Figure 4. Key biological insights that have been consistently observed following the application of proteogenomics to multiple tumor types include (*a*) the addition of phosphoproteomics, which provided a more detailed characterization of downstream signaling pathways beyond the driving mutation, identifying potential alternative therapeutic targets (8, 11, 14, 15, 17, 19, 36, 37); (*b*) stratification of tumors into immune-hot and immune-cold subtypes, which provided insights into factors that potentially modify the response to immunotherapy (16, 17, 43, 60, 63); and (*c*) identification of consistently discordant mRNA-protein pairs, which implicated translational regulation and protein degradation as important components of cancer biology (7–17, 167).

To further evolve the field of proteogenomics, more advances will be needed in several key areas.

### Scaling Down: Miniaturizing Inputs

It will be important to harness and develop new technologies that facilitate testing of smaller amounts of input material (168). This is critical for several important reasons. First, primary tumors are often difficult to obtain and material is limited, especially in disease relapse stages for which there is a critical dearth of knowledge. Second, tumors are highly heterogeneous, and it is important to study each cellular component. Fractionation of these cell subpopulations often yields scarce numbers of cells for analysis. Finally, the capacity to study biology at a single-cell or near-single-cell level is pushing new frontiers in nucleic acid sequencing and promises to do the same for proteomics (including PTMs) (126, 169, 170) and/or metabolomics (171–173).

### Scaling Up: Multiplexing

As noted above, the capacity for multiplexing samples has and continues to improve (71, 174–176). This has come with great benefits for reducing costs and minimizing batch effects. In addition to the intratumoral heterogeneity noted above, there is also extreme heterogeneity across patients. As such, it is critical to develop multiplexing techniques and economies of scale to enable proteomic analysis of larger numbers of patient tumors. In this way, identification of proteomic patterns of tumor groups with less common clinical or genetic profiles, which collectively encompass large proportions of tumors, will be enabled. In addition, the breadth of PTM analytes that are measured by MS has increased dramatically (77, 177, 178), and it will be important to continue this trajectory to better understand the biology and clinical ramifications of these important protein modifications.

### Dynamic Measurements

Nearly all proteomic data collection to date has been performed at static, baseline conditions. While this is a critical first step toward establishing the proteomic landscape of each tumor, it is also clear that tumors respond to therapeutic stress in diverse ways. Hence, it will only be through longitudinal and dose-dependent testing of dynamic changes that occur after patients have been treated with therapeutic regimens that we will be able to fully harness proteogenomic data for diagnostic, prognostic, and therapeutic deliverables (179). It may be possible to obtain some of this dynamic information through short-term, ex vivo exposure of primary tumor specimens to panels of agents, which will offer information on larger numbers of potential therapeutics than would be clinically feasible. The increasing sensitivity and throughput of MS proteomics approaches discussed above will be critical to accomplishing this goal.

### Translating the Work

The proteogenomic analysis of diverse tumor types has yielded a wealth of findings, many of which point to potential new therapeutic strategies and/or new mechanistic insights that should be further pursued. Proteogenomics itself may also offer new diagnostic platforms to help guide when and where therapies are deployed. Continued progress in these areas will be essential for the ultimate fulfillment of the translational promise of proteogenomics.

Collectively, a great deal has been accomplished and learned through proteogenomic analyses of primary tumors. Through continued efforts along the same lines as well as expansion into the areas noted above, the future looks bright for proteogenomics to continue having a major impact on our knowledge of tumor biology and clinical care of patients with cancer.

## DISCLOSURE STATEMENT

B.J.D. has served on the scientific advisory board of Adela Bio, Aileron Therapeutics, Therapy Architects/ALLCRON (inactive), Cepheid, Celgene, DNA SEQ, Nemucore Medical Innovations, Novartis, RUNX1 Research Program, and Vivid Biosciences (inactive); has served on the scientific advisory board of and holds stock in Aptose Biosciences, Blueprint Medicines, Enliven Therapeutics, Iterion Therapeutics, GRAIL, and Recludix Pharma; has served on the Board of Directors for and holds stock in Amgen and Vincerx Pharma; has served on the Board of Directors for Burroughs Wellcome Fund and CureOne; has served on the joint steering committee of the Leukemia & Lymphoma Society’s Beat AML; has served on the advisory committee of the Multicancer Early Detection Consortium; is a founder of VB Therapeutics; has a sponsored research agreement with Enliven Therapeutics and Recludix Pharma; has clinical trial funding from Novartis and Astra-Zeneca; receives royalties from US Patent 6,958,335 (Novartis exclusive license) and from OHSU and Dana-Farber Cancer Institute (one Merck exclusive license, one CytoImage, Inc., exclusive license, and one Sun Pharma Advanced Research Company nonexclusive license); and holds US Patents 4,326,534, 6,958,335, 7,416,873, 7,592,142, 10,473,667, 10,664,967, and 11,049,247. J.W.T. received research support from Acerta Pharma, Agios Pharmaceuticals, Aptose Biosciences, Array BioPharma, AstraZeneca, Constellation Pharmaceuticals, Genentech, Gilead Sciences, Incyte, Janssen Pharmaceuticals, Kronos Bio, Meryx, Petra Pharma, Schrödinger, Seattle Genetics, Syros Pharmaceuticals, Takeda Pharmaceuticals, and Tolero Pharmaceuticals and serves on the advisory board for Recludix Pharma. C.E.T. served on the scientific advisory board of and had research sponsored by Notable Labs and has served as a consultant for St. Jude’s Children’s Hospital.

## Figures and Tables

**Table 1:** Multiple tumor types that have been profiled through the lens of proteomics

Cancer type	Patient cohort size	Protein data type(s)	Biological insights	PMID	Year	Reference
ALL	*N* = 27	Global	CTCF and cohesion under expression in ETV6/RUNX1-positive, hyperdiploid pediatric ALL	30944321	2019	42
AML	*N* = 20	Global, phospho	Development of KSEA using AML model	23532336	2013	31
	*N* = 21	Global, phospho	Phosphoproteomic signature predicting FLT3 inhibitor response	24247654	2014	33
	*N* = 30	Global, phospho	CD marker evaluation identified importance of increased MEK and PKC pathway activity for AML differentiation	29626197	2018	32
	*N* = 9	Global, phospho	Phosphoproteomic signature of pacritinib sensitivity uncovered important pathways downstream of IRAK1	29743719	2018	34
	*N* = 42	Global	Plasma membrane proteomics identified 50 leukemia-enriched proteins, which enabled characterization of functionally distinct subclones of AML	30245083	2018	54
	*N* = 15	Global	Nuclear proteomics revealed S100A4 as a potential therapeutic target in AML	31611628	2020	55
	*N* = 14	Global	Comprehensive proteomic and transcriptomic resource of functionally characterized and validated AML LSCs, blasts, and healthy HSPCs	32556243	2020	40
	*N* = 7	Global, phospho	Phosphoproteomic profiling of wild-type and mutant FLT3 AML patient samples uncovered synergy between DNA-PK and FLT3 inhibitors	33067575	2021	36
	*N* = 33	Global, phospho	Cellular aging influences cytoskeletal functioning in chemoresistant AML cells, which may influence mitosis, polarity, intracellular transport, and adhesion	33349623	2020	29
	*N* = 11	Global	Early FLT3 inhibitor–resistant cells depend upon AURKB and undergo metabolic reprogramming versus late resistant cells that expand preexisting NRAS mutant subclones	34171263	2021	37
	*N* = 252	Global, phospho	Identification of a proteomic subtype of AML characterized by high expression of mitochondrial proteins (Mito-AML) and poor outcome	35245447	2022	28
	*N* = 6	Global	Gilteritinib found to synergize with venetoclax, and proteomics revealed increased FLT3 wild-type signaling in specimens with low in vitro response to the currently used venetoclax-azacitidine combination	35857899	2022	35
	*N* = 44	Global, phospho	Orthogonal validation of deep-scale proteome and phosphoproteome database from representative AML patients	35895896	2022	30
	*N* = 38	Global, phospho	Proteomic and phosphoproteomic signatures have potential in stratifying patient drug sensitivities for AML	35896960	2022	39
	*N* = 20	Global, phospho	Primary AML phosphoproteomics reveals rationale for targeting AKT in the context of functional p53 to overcome selinexor resistance	35947955	2022	53
	*N* = 11	Global, phospho	AKT-mTORC1-ULK1-dependent autophagy was identified as a dominant resistance mechanism to on-target FLT3 inhibitor therapy	35999260	2022	38
Bile duct	*N* = 262	Global, phospho	TP53 and KRAS contribute to metastasis, FGFR2 fusions activate Rho GTPase pathway, and SLC16A3 and HKDC1 are potential prognostic biomarkers	34971568	2022	67
Brain	*N* = 218 pediatric	Global, phospho	Similarities between some craniopharyngioma and low-grade glioma with *BRAF* V600E mutations	33242424	2020	18
	*N* = 99 GBM	Global, phospho, acetyl, metabolomics	RTKs, PTPN1, and PLCG1 signaling hubs; different immune subtypes	33577785	2021	17
Breast	*N* = 105	Global, phospho	CDK12, PAK1, PTK2, RIPK2, and TLK2	27251275	2016	8
	*N* = 122	Global, phospho, acetyl	*STAT1* and *IFNG* potential markers for immunotherapy; Rb protein phosphorylation correlated with response to CDK4/6	33212010	2020	11
Colorectal	*N* = 95	Global	*HNF4A*, *SRC*, and *TOMM34*	25043054	2014	7
	*N* = 110	Global, phospho	Rb phosphorylation and glycolysis dependence	31031003	2019	10
Endometrial	*N* = 95	Global, phospho, acetyl	Protein signatures correlated with histologic subtypes; regulation of EMT by QK1	32059776	2020	14
Gastric	*N* = 80	Global, phospho, glyco	Four subtypes associated with proliferation, immune response, metabolism, and invasion	30645970	2019	63
HNSCC	*N* = 50	Global	Polymorphism in APOBEC3 and increased expression of A3A has clinical prognostic relevance	28878238	2017	62
	*N* = 108	Global, phospho	Protein expression of EGFR ligands is a more predictive response to EGFR therapy; HPV-negative HNSCC is immune cold	33417831	2021	19
Kidney	*N* = 103	Global, phospho	Hypoxia, glycolysis, EMT, and inflammation with downregulation of oxidative phosphorylation	31675502	2019	13
	*N* = 213	Global, phospho, glyco, metabolomics	Molecular features correlated with histologic subtypes	36563681	2023	61
Liver	*N* = 159	Global, phospho	Three proteomic subgroups; PYCR2 and ADH1A biomarkers; CTNNB1 and TP53 mutations associated with glycolysis and cell proliferation	31585088	2019	64
Lung	*N* = 110, LUAD	Global, phospho, acetyl	SOS1 in *KRAS* mutant; PTPN11 in *ALK* and *EGFR* mutant; *STK11* mutations with immune-cold tumors	32649874	2020	15
	*N* = 103	Global, phospho	APOBEC signature in younger women; early-stage EGFR-mutated disease identified by proteomics	32649875	2020	65
	*N* = 108, LSCC	Global, phospho, acetyl, ubiquityl	Unique subtypes associated with EMT and phosphorylation signatures; immune signatures	34358469	2021	16
Melanoma	*N* = 505	Global, phospho, acetyl	Proteomic features not predicted by genome sequencing alone	34323402	2021	66
Multiple myeloma	*N* = 5	Global, phospho	CDK6 resistance signature that included TRIP13 and RRM1; CDK6 inhibitors synergistic with immunomodulatory drugs	35197447	2022	43
Ovarian	*N* = 174	Global, phospho, acetyl	Proteins enriched in cell motility, invasion, and immune regulation	27372738	2016	9
	*N* = 83	Global, phospho	Mitotic and replicative stress	32529193	2020	12
	*N* = 83	Global, glyco	Tumor-specific glycosylation	33086064	2020	59
Pancreas	*N* = 140	Global, phospho, glyco	Glycoprotein expression and *KRAS* mutations; identification of immune-cold PDAC tumors	34534465	2021	60
Prostate	*N* = 38	Global	MicroRNA target correlations identified at protein, but not messenger RNA, level; metabolic shifts in the citric acid cycle during progression	29563510	2018	167
	*N* = 17	Global	Decreased REST and TP53 leads to neuroendocrine features	30274982	2019	180
	*N* = 76	Global, acetyl	Five proteomic subtypes; prognostic biomarkers combining genomic, epigenomic, and proteomic data outperform single data type	30889379	2019	181

These studies not only provided biological insights but also established common statistical methodologies to systematically process the amassing omics data sets. Full data sets (with accession numbers) and information of patient cohorts are available online and can be accessed via the provided PMID and reference numbers. Abbreviations: ALL, acute lymphoblastic leukemia; AML, acute myeloid leukemia; CDK, cyclin-dependent kinase; CTCF, CCCTC-binding factor; EGFR, epidermal growth factor receptor; EMT, epithelial-mesenchymal transition; FGFR2, fibroblast growth factor receptor 2; GBM, glioblastoma multiforme; HNSCC, head and neck squamous cell carcinoma; HPV, human papilloma virus; KSEA, kinase-substrate enrichment analysis; LSC, leukemic stem cell; LSCC, lung squamous cell carcinoma; LUAD, lung adenocarcinoma; PDAC, pancreatic ductal carcinoma; PKC, protein kinase C; PLCG1, phospholipase C gamma 1; PMID, PubMed ID; PTPN, protein tyrosine phosphatase non-receptor type 1; RRM1, ribonucleotide reductase catalytic subunit M1; RTK, receptor tyrosine kinase; TRIP13, thyroid hormone receptor interactor 13.

## References

[1] Druker BJ, Tamura S, Buchdunger E, Ohno S, Segal GM, 1996. Effects of a selective inhibitor of the Abl tyrosine kinase on the growth of Bcr-Abl positive cells. Nat. Med 2:561–66 8616716 10.1038/nm0596-561

[2] Druker BJ, Talpaz M, Resta DJ, Peng B, Buchdunger E, 2001. Efficacy and safety of a specific inhibitor of the BCR-ABL tyrosine kinase in chronic myeloid leukemia. N. Engl. J. Med 344:1031–37 11287972 10.1056/NEJM200104053441401

[3] Slamon DJ, Leyland-Jones B, Shak S, Fuchs H, Paton V, 2001. Use of chemotherapy plus a monoclonal antibody against HER2 for metastatic breast cancer that overexpresses HER2. N. Engl. J. Med 344:783–92 11248153 10.1056/NEJM200103153441101

[4] Cooper LA, Demicco EG, Saltz JH, Powell RT, Rao A, Lazar AJ. 2018. PanCancer insights from The Cancer Genome Atlas: the pathologist’s perspective. J. Pathol 244:512–24 29288495 10.1002/path.5028 PMC6240356

[5] Wang Z, Jensen MA, Zenklusen JC. 2016. A practical guide to The Cancer Genome Atlas (TCGA). Methods Mol. Biol 1418:111–41 27008012 10.1007/978-1-4939-3578-9_6

[6] Mani DR, Krug K, Zhang B, Satpathy S, Clauser KR, 2022. Cancer proteogenomics: current impact and future prospects. Nat. Rev. Cancer 22:298–313 35236940 10.1038/s41568-022-00446-5 PMC12404316

[7] Zhang B, Wang J, Wang X, Zhu J, Liu Q, 2014. Proteogenomic characterization of human colon and rectal cancer. Nature 513:382–87 25043054 10.1038/nature13438 PMC4249766

[8] Mertins P, Mani DR, Ruggles KV, Gillette MA, Clauser KR, 2016. Proteogenomics connects somatic mutations to signalling in breast cancer. Nature 534:55–62 27251275 10.1038/nature18003 PMC5102256

[9] Zhang H, Liu T, Zhang Z, Payne SH, Zhang B, 2016. Integrated proteogenomic characterization of human high-grade serous ovarian cancer. Cell 166:755–65 27372738 10.1016/j.cell.2016.05.069 PMC4967013

[10] Vasaikar S, Huang C, Wang X, Petyuk VA, Savage SR, 2019. Proteogenomic analysis of human colon cancer reveals new therapeutic opportunities. Cell 177:1035–49.e19 31031003 10.1016/j.cell.2019.03.030 PMC6768830

[11] Krug K, Jaehnig EJ, Satpathy S, Blumenberg L, Karpova A, 2020. Proteogenomic landscape of breast cancer tumorigenesis and targeted therapy. Cell 183:1436–56.e31 33212010 10.1016/j.cell.2020.10.036 PMC8077737

[12] McDermott JE, Arshad OA, Petyuk VA, Fu Y, Gritsenko MA, 2020. Proteogenomic characterization of ovarian HGSC implicates mitotic kinases, replication stress in observed chromosomal instability. Cell Rep. Med 1:100004 32529193 10.1016/j.xcrm.2020.100004 PMC7289043

[13] Clark DJ, Dhanasekaran SM, Petralia F, Pan J, Song X, 2019. Integrated proteogenomic characterization of clear cell renal cell carcinoma. Cell 179:964–83.e31 31675502 10.1016/j.cell.2019.10.007 PMC7331093

[14] Dou Y, Kawaler EA, Cui Zhou D, Gritsenko MA, Huang C, 2020. Proteogenomic characterization of endometrial carcinoma. Cell 180:729–48.e26 32059776 10.1016/j.cell.2020.01.026 PMC7233456

[15] Gillette MA, Satpathy S, Cao S, Dhanasekaran SM, Vasaikar SV, 2020. Proteogenomic characterization reveals therapeutic vulnerabilities in lung adenocarcinoma. Cell 182:200–25.e35 32649874 10.1016/j.cell.2020.06.013 PMC7373300

[16] Satpathy S, Krug K, Jean Beltran PM, Savage SR, Petralia F, 2021. A proteogenomic portrait of lung squamous cell carcinoma. Cell 184:4348–71.e40 34358469 10.1016/j.cell.2021.07.016 PMC8475722

[17] Wang LB, Karpova A, Gritsenko MA, Kyle JE, Cao S, 2021. Proteogenomic and metabolomic characterization of human glioblastoma. Cancer Cell 39:509–28.e20 33577785 10.1016/j.ccell.2021.01.006 PMC8044053

[18] Petralia F, Tignor N, Reva B, Koptyra M, Chowdhury S, 2020. Integrated proteogenomic characterization across major histological types of pediatric brain cancer. Cell 183:1962–85.e31 33242424 10.1016/j.cell.2020.10.044 PMC8143193

[19] Huang C, Chen L, Savage SR, Eguez RV, Dou Y, 2021. Proteogenomic insights into the biology and treatment of HPV-negative head and neck squamous cell carcinoma. Cancer Cell 39:361–79.e16 33417831 10.1016/j.ccell.2020.12.007 PMC7946781

[20] Bottomly D, Long N, Schultz AR, Kurtz SE, Tognon CE, 2022. Integrative analysis of drug response and clinical outcome in acute myeloid leukemia. Cancer Cell 40:850–64.e9 35868306 10.1016/j.ccell.2022.07.002 PMC9378589

[21] Hochhaus A, Larson RA, Guilhot F, Radich JP, Branford S, 2017. Long-term outcomes of imatinib treatment for chronic myeloid leukemia. N. Engl. J. Med 376:917–27 28273028 10.1056/NEJMoa1609324 PMC5901965

[22] Ley TJ, Miller C, Ding L, Raphael BJ, Mungall AJ, 2013. Genomic and epigenomic landscapes of adult de novo acute myeloid leukemia. N. Engl. J. Med 368:2059–74 23634996 10.1056/NEJMoa1301689 PMC3767041

[23] Kumar CC. 2011. Genetic abnormalities and challenges in the treatment of acute myeloid leukemia. Genes Cancer 2:95–107 21779483 10.1177/1947601911408076 PMC3111245

[24] Papaemmanuil E, Gerstung M, Bullinger L, Gaidzik VI, Paschka P, 2016. Genomic classification and prognosis in acute myeloid leukemia. N. Engl. J. Med 374:2209–21 27276561 10.1056/NEJMoa1516192 PMC4979995

[25] Tyner JW, Tognon CE, Bottomly D, Wilmot B, Kurtz SE, 2018. Functional genomic landscape of acute myeloid leukaemia. Nature 562:526–31 30333627 10.1038/s41586-018-0623-z PMC6280667

[26] Yang F, Long N, Anekpuritanang T, Bottomly D, Savage JC, 2022. Identification and prioritization of myeloid malignancy germline variants in a large cohort of adult patients with AML. Blood 139:1208–21 34482403 10.1182/blood.2021011354 PMC9211447

[27] Joshi SK, Tognon CE, Druker BJ, Rodland KD. 2023. Oncoproteomic profiling of AML: moving beyond genomics. Expert Rev. Proteom 19:283–87 10.1080/14789450.2023.2176757 PMC10505090 36734985

[28] Jayavelu AK, Wolf S, Buettner F, Alexe G, Haupl B, 2022. The proteogenomic subtypes of acute myeloid leukemia. Cancer Cell 40:301–17.e12 35245447 10.1016/j.ccell.2022.02.006 PMC12882723

[29] Hernandez-Valladares M, Aasebo E, Berven F, Selheim F, Bruserud O. 2020. Biological characteristics of aging in human acute myeloid leukemia cells: the possible importance of aldehyde dehydrogenase, the cytoskeleton and altered transcriptional regulation. Aging 12:24734–77 33349623 10.18632/aging.202361 PMC7803495

[30] Kramer MH, Zhang Q, Sprung R, Day RB, Erdmann-Gilmore P, 2022. Proteomic and phosphoproteomic landscapes of acute myeloid leukemia. Blood 140:1533–48 35895896 10.1182/blood.2022016033 PMC9523374

[31] Casado P, Rodriguez-Prados JC, Cosulich SC, Guichard S, Vanhaesebroeck B, 2013. Kinase-substrate enrichment analysis provides insights into the heterogeneity of signaling pathway activation in leukemia cells. Sci. Signal 6:rs6 10.1126/scisignal.2003573 23532336

[32] Casado P, Wilkes EH, Miraki-Moud F, Hadi MM, Rio-Machin A, 2018. Proteomic and genomic integration identifies kinase and differentiation determinants of kinase inhibitor sensitivity in leukemia cells. Leukemia 32:1818–22 29626197 10.1038/s41375-018-0032-1 PMC5949212

[33] Schaab C, Oppermann FS, Klammer M, Pfeifer H, Tebbe A, 2014. Global phosphoproteome analysis of human bone marrow reveals predictive phosphorylation markers for the treatment of acute myeloid leukemia with quizartinib. Leukemia 28:716–19 24247654 10.1038/leu.2013.347 PMC3948157

[34] Hosseini MM, Kurtz SE, Abdelhamed S, Mahmood S, Davare MA, 2018. Inhibition of interleukin-1 receptor-associated kinase-1 is a therapeutic strategy for acute myeloid leukemia subtypes. Leukemia 32:2374–87 29743719 10.1038/s41375-018-0112-2 PMC6558520

[35] Janssen M, Schmidt C, Bruch PM, Blank MF, Rohde C, 2022. Venetoclax synergizes with gilteritinib in FLT3 wildtype high-risk acute myeloid leukemia by suppressing MCL-1. Blood 140:2594–610 35857899 10.1182/blood.2021014241

[36] Murray HC, Enjeti AK, Kahl RGS, Flanagan HM, Sillar J, 2021. Quantitative phosphoproteomics uncovers synergy between DNA-PK and FLT3 inhibitors in acute myeloid leukaemia. Leukemia 35:1782–87 33067575 10.1038/s41375-020-01050-y PMC8179851

[37] Joshi SK, Nechiporuk T, Bottomly D, Piehowski PD, Reisz JA, 2021. The AML microenvironment catalyzes a stepwise evolution to gilteritinib resistance. Cancer Cell 39:999–1014.e8 34171263 10.1016/j.ccell.2021.06.003 PMC8686208

[38] Koschade SE, Klann K, Shaid S, Vick B, Stratmann JA, 2022. Translatome proteomics identifies autophagy as a resistance mechanism to on-target FLT3 inhibitors in acute myeloid leukemia. Leukemia 36:2396–407 35999260 10.1038/s41375-022-01678-y PMC9522593

[39] Gosline SJC, Tognon C, Nestor M, Joshi S, Modak R, 2022. Proteomic and phosphoproteomic measurements enhance ability to predict ex vivo drug response in AML. Clin. Proteom 19:30 10.1186/s12014-022-09367-9 PMC9327422 35896960

[40] Raffel S, Klimmeck D, Falcone M, Demir A, Pouya A, 2020. Quantitative proteomics reveals specific metabolic features of acute myeloid leukemia stem cells. Blood 136:1507–19 32556243 10.1182/blood.2019003654

[41] Stratmann S, Vesterlund M, Umer HM, Eshtad S, Skaftason A, 2022. Proteogenomic analysis of acute myeloid leukemia associates relapsed disease with reprogrammed energy metabolism both in adults and children. Leukemia 37:550–59 36572751 10.1038/s41375-022-01796-7 PMC9991901

[42] Yang M, Vesterlund M, Siavelis I, Moura-Castro LH, Castor A, 2019. Proteogenomics and Hi-C reveal transcriptional dysregulation in high hyperdiploid childhood acute lymphoblastic leukemia. Nat. Commun 10:1519 30944321 10.1038/s41467-019-09469-3 PMC6447538

[43] Ng YLD, Ramberger E, Bohl SR, Dolnik A, Steinebach C, 2022. Proteomic profiling reveals CDK6 upregulation as a targetable resistance mechanism for lenalidomide in multiple myeloma. Nat. Commun 13:1009 35197447 10.1038/s41467-022-28515-1 PMC8866544

[44] Aaseb E, Berven FS, Bartaula-Brevik S, Stokowy T, Hovland R, 2020. Proteome and phosphoproteome changes associated with prognosis in acute myeloid leukemia. Cancers 12:709 32192169 10.3390/cancers12030709 PMC7140113

[45] Hernandez-Valladares M, Bruserud O, Selheim F. 2020. The implementation of mass spectrometry-based proteomics workflows in clinical routines of acute myeloid leukemia: applicability and perspectives. Int. J. Mol. Sci 21:6830 32957646 10.3390/ijms21186830 PMC7556012

[46] Segura V, Valero ML, Cantero L, Munoz J, Zarzuela E, 2018. In-depth proteomic characterization of classical and non-classical monocyte subsets. Proteomes 6:8 29401756 10.3390/proteomes6010008 PMC5874767

[47] Kwon YW, Jo HS, Bae S, Seo Y, Song P, 2021. Application of proteomics in cancer: recent trends and approaches for biomarkers discovery. Front. Med 8:747333 10.3389/fmed.2021.747333 PMC8492935 34631760

[48] Samuel G, Crow J, Klein JB, Merchant ML, Nissen E, 2020. Ewing sarcoma family of tumors-derived small extracellular vesicle proteomics identify potential clinical biomarkers. Oncotarget 11:2995–3012 32821345 10.18632/oncotarget.27678 PMC7415402

[49] Rontogianni S, Synadaki E, Li B, Liefaard MC, Lips EH, 2019. Proteomic profiling of extracellular vesicles allows for human breast cancer subtyping. Commun. Biol 2:325 31508500 10.1038/s42003-019-0570-8 PMC6722120

[50] Ponzini E, Santambrogio C, De Palma A, Mauri P, Tavazzi S, Grandori R. 2022. Mass spectrometry-based tear proteomics for noninvasive biomarker discovery. Mass Spectrom. Rev 41:842–60 33759206 10.1002/mas.21691 PMC9543345

[51] Schmid D, Warnken U, Latzer P, Hoffmann DC, Roth J, 2021. Diagnostic biomarkers from proteomic characterization of cerebrospinal fluid in patients with brain malignancies. J. Neurochem 158:522–38 33735443 10.1111/jnc.15350

[52] Schoof EM, Furtwangler B, Uresin N, Rapin N, Savickas S, 2021. Quantitative single-cell proteomics as a tool to characterize cellular hierarchies. Nat. Commun 12:3341 34099695 10.1038/s41467-021-23667-y PMC8185083

[53] Emdal KB, Palacio-Escat N, Wigerup C, Eguchi A, Nilsson H, 2022. Phosphoproteomics of primary AML patient samples reveals rationale for AKT combination therapy and p53 context to overcome selinexor resistance. Cell Rep. 40:111177 35947955 10.1016/j.celrep.2022.111177 PMC9380259

[54] de Boer B, Prick J, Pruis MG, Keane P, Imperato MR, 2018. Prospective isolation and characterization of genetically and functionally distinct AML subclones. Cancer Cell 34:674–89.e8 30245083 10.1016/j.ccell.2018.08.014

[55] Alanazi B, Munje CR, Rastogi N, Williamson AJK, Taylor S, 2020. Integrated nuclear proteomics and transcriptomics identifies S100A4 as a therapeutic target in acute myeloid leukemia. Leukemia 34:427–40 31611628 10.1038/s41375-019-0596-4 PMC6995695

[56] Kang KW, Kim H, Hur W, Jung JH, Jeong SJ, 2021. A proteomic approach to understand the clinical significance of acute myeloid leukemia-derived extracellular vesicles reflecting essential characteristics of leukemia. Mol. Cell. Proteom 20:100017 10.1074/mcp.RA120.002169 PMC7949255 33592500

[57] Mistry AM, Greenplate AR, Ihrie RA, Irish JM. 2019. Beyond the message: advantages of snapshot proteomics with single-cell mass cytometry in solid tumors. FEBS J 286:1523–39 30549207 10.1111/febs.14730 PMC6478512

[58] Watson J, Ferguson HR, Brady RM, Ferguson J, Fullwood P, 2022. Spatially resolved phosphoproteomics reveals fibroblast growth factor receptor recycling-driven regulation of autophagy and survival. Nat. Commun 13:6589 36329028 10.1038/s41467-022-34298-2 PMC9633600

[59] Hu Y, Pan J, Shah P, Ao M, Thomas SN, 2020. Integrated proteomic and glycoproteomic characterization of human high-grade serous ovarian carcinoma. Cell Rep. 33:108276 33086064 10.1016/j.celrep.2020.108276 PMC7970828

[60] Cao L, Huang C, Zhou DC, Hu Y, Lih TM, 2021. Proteogenomic characterization of pancreatic ductal adenocarcinoma. Cell 184:5031–52.e26 34534465 10.1016/j.cell.2021.08.023 PMC8654574

[61] Li Y, Lih TM, Dhanasekaran SM, Mannan R, Chen L, 2023. Histopathologic and proteogenomic heterogeneity reveals features of clear cell renal cell carcinoma aggressiveness. Cancer Cell 41:139–63.e17 36563681 10.1016/j.ccell.2022.12.001 PMC9839644

[62] Chen TW, Lee CC, Liu H, Wu CS, Pickering CR, 2017. APOBEC3A is an oral cancer prognostic biomarker in Taiwanese carriers of an APOBEC deletion polymorphism. Nat. Commun 8:465 28878238 10.1038/s41467-017-00493-9 PMC5587710

[63] Mun DG, Bhin J, Kim S, Kim H, Jung JH, 2019. Proteogenomic characterization of human early-onset gastric cancer. Cancer Cell 35:111–24.e10 30645970 10.1016/j.ccell.2018.12.003

[64] Gao Q, Zhu H, Dong L, Shi W, Chen R, 2019. Integrated proteogenomic characterization of HBV-related hepatocellular carcinoma. Cell 179:561–77.e22 31585088 10.1016/j.cell.2019.08.052

[65] Chen YJ, Roumeliotis TI, Chang YH, Chen CT, Han CL, 2020. Proteogenomics of non-smoking lung cancer in East Asia delineates molecular signatures of pathogenesis and progression. Cell 182:226–44.e17 32649875 10.1016/j.cell.2020.06.012

[66] Betancourt LH, Gil J, Sanchez A, Doma V, Kuras M, 2021. The Human Melanoma Proteome Atlas—complementing the melanoma transcriptome. Clin. Transl. Med 11:e451 34323402 10.1002/ctm2.451 PMC8299047

[67] Dong L, Lu D, Chen R, Lin Y, Zhu H, 2022. Proteogenomic characterization identifies clinically relevant subgroups of intrahepatic cholangiocarcinoma. Cancer Cell 40:70–87.e15 34971568 10.1016/j.ccell.2021.12.006

[68] Ross PL, Huang YN, Marchese JN, Williamson B, Parker K, 2004. Multiplexed protein quantitation in *Saccharomyces cerevisiae* using amine-reactive isobaric tagging reagents. Mol. Cell. Proteom 3:1154–69 10.1074/mcp.M400129-MCP200 15385600

[69] McAlister GC, Huttlin EL, Haas W, Ting L, Jedrychowski MP, 2012. Increasing the multiplexing capacity of TMTs using reporter ion isotopologues with isobaric masses. Anal. Chem 84:7469–78 22880955 10.1021/ac301572t PMC3715028

[70] Thompson A, Wolmer N, Koncarevic S, Selzer S, Bohm G, 2019. TMTpro: design, synthesis, and initial evaluation of a proline-based isobaric 16-plex tandem mass tag reagent set. Anal. Chem 91:15941–50 31738517 10.1021/acs.analchem.9b04474

[71] Li J, Cai Z, Bomgarden RD, Pike I, Kuhn K, 2021. TMTpro-18plex: the expanded and complete set of TMTpro reagents for sample multiplexing. J. Proteome Res 20:2964–72 33900084 10.1021/acs.jproteome.1c00168 PMC8210943

[72] Sun H, Poudel S, Vanderwall D, Lee DG, Li Y, Peng J. 2022. 29-Plex tandem mass tag mass spectrometry enabling accurate quantification by interference correction. Proteomics 22:e2100243 35723178 10.1002/pmic.202100243 PMC9588555

[73] Gillet LC, Navarro P, Tate S, Rost H, Selevsek N, 2012. Targeted data extraction of the MS/MS spectra generated by data-independent acquisition: a new concept for consistent and accurate proteome analysis. Mol. Cell. Proteom 11:O111.016717 10.1074/mcp.O111.016717 PMC3433915 22261725

[74] Mertins P, Tang LC, Krug K, Clark DJ, Gritsenko MA, 2018. Reproducible workflow for multiplexed deep-scale proteome and phosphoproteome analysis of tumor tissues by liquid chromatography-mass spectrometry. Nat. Protoc 13:1632–61 29988108 10.1038/s41596-018-0006-9 PMC6211289

[75] Udeshi ND, Mani DC, Satpathy S, Fereshetian S, Gasser JA, 2020. Rapid and deep-scale ubiquitylation profiling for biology and translational research. Nat. Commun 11:359 31953384 10.1038/s41467-019-14175-1 PMC6969155

[76] Gajadhar AS, Johnson H, Slebos RJ, Shaddox K, Wiles K, 2015. Phosphotyrosine signaling analysis in human tumors is confounded by systemic ischemia-driven artifacts and intra-specimen heterogeneity. Cancer Res. 75:1495–503 25670172 10.1158/0008-5472.CAN-14-2309 PMC4383696

[77] Abelin JG, Bergstrom EJ, Taylor HB, Rivera KD, Klaeger S, 2022. MONTE enables serial immunopeptidome, ubiquitylome, proteome, phosphoproteome, acetylome analyses of sample-limited tissues. bioRxiv 2021.06.22.449417. 10.1101/2021.06.22.449417 PMC10070353 37012232

[78] van Bentum M, Selbach M. 2021. An introduction to advanced targeted acquisition methods. Mol. Cell. Proteom 20:100165 10.1016/j.mcpro.2021.100165 PMC8600983 34673283

[79] Lange V, Picotti P, Domon B, Aebersold R. 2008. Selected reaction monitoring for quantitative proteomics: a tutorial. Mol. Syst. Biol 4:222 18854821 10.1038/msb.2008.61 PMC2583086

[80] Peterson AC, Russell JD, Bailey DJ, Westphall MS, Coon JJ. 2012. Parallel reaction monitoring for high resolution and high mass accuracy quantitative, targeted proteomics. Mol. Cell. Proteom 11:1475–88 10.1074/mcp.O112.020131 PMC3494192 22865924

[81] Gallien S, Duriez E, Crone C, Kellmann M, Moehring T, Domon B. 2012. Targeted proteomic quantification on quadrupole-orbitrap mass spectrometer. Mol. Cell. Proteom 11:1709–23 10.1074/mcp.O112.019802 PMC3518128 22962056

[82] Gallien S, Kim SY, Domon B. 2015. Large-scale targeted proteomics using internal standard triggered-parallel reaction monitoring (IS-PRM). Mol. Cell. Proteom 14:1630–44 10.1074/mcp.O114.043968 PMC4458725 25755295

[83] Mao Y, Wang X, Huang P, Tian R. 2021. Spatial proteomics for understanding the tissue microenvironment. Analyst 146:3777–98 34042124 10.1039/d1an00472g

[84] Mund A, Brunner AD, Mann M. 2022. Unbiased spatial proteomics with single-cell resolution in tissues. Mol. Cell 82:2335–49 35714588 10.1016/j.molcel.2022.05.022

[85] Piehowski PD, Zhu Y, Bramer LM, Stratton KG, Zhao R, 2020. Automated mass spectrometry imaging of over 2000 proteins from tissue sections at 100-mm spatial resolution. Nat. Commun 11:8 31911630 10.1038/s41467-019-13858-z PMC6946663

[86] Swensen AC, Velickovic D, Williams SM, Moore RJ, Day LZ, 2022. Proteomic profiling of intra-islet features reveals substructure-specific protein signatures. Mol. Cell. Proteom 21:100426 10.1016/j.mcpro.2022.100426 PMC9706166 36244662

[87] Gosline SJ, Velickovic M, Pino J, Day LZ, Attah IK, 2022. Proteome mapping of the human pancreatic islet microenvironment reveals endocrine-exocrine signaling sphere of influence. bioRxiv 2022.11.21.517388. 10.1101/2022.11.21.517388 PMC10460696 37328065

[88] Sharma K, Schmitt S, Bergner CG, Tyanova S, Kannaiyan N, 2015. Cell type- and brain region-resolved mouse brain proteome. Nat. Neurosci 18:1819–31 26523646 10.1038/nn.4160 PMC7116867

[89] Wisniewski JR, Ostasiewicz P, Mann M. 2011. High recovery FASP applied to the proteomic analysis of microdissected formalin fixed paraffin embedded cancer tissues retrieves known colon cancer markers. J. Proteome Res 10:3040–49 21526778 10.1021/pr200019m

[90] Carlyle BC, Kitchen RR, Kanyo JE, Voss EZ, Pletikos M, 2017. A multiregional proteomic survey of the postnatal human brain. Nat. Neurosci 20:1787–95 29184206 10.1038/s41593-017-0011-2 PMC5894337

[91] Doll S, Dressen M, Geyer PE, Itzhak DN, Braun C, 2017. Region and cell-type resolved quantitative proteomic map of the human heart. Nat. Commun 8:1469 29133944 10.1038/s41467-017-01747-2 PMC5684139

[92] Huang P, Kong Q, Gao W, Chu B, Li H, 2020. Spatial proteome profiling by immunohistochemistry-based laser capture microdissection and data-independent acquisition proteomics. Anal. Chim. Acta 1127:140–48 32800117 10.1016/j.aca.2020.06.049

[93] Griesser E, Wyatt H, Ten Have S, Stierstorfer B, Lenter M, Lamond AI. 2020. Quantitative profiling of the human substantia nigra proteome from laser-capture microdissected FFPE tissue. Mol. Cell. Proteom 19:839–51 10.1074/mcp.RA119.001889 PMC7196589 32132230

[94] Clair G, Piehowski PD, Nicola T, Kitzmiller JA, Huang EL, 2016. Spatially-resolved proteomics: rapid quantitative analysis of laser capture microdissected alveolar tissue samples. Sci. Rep 6:39223 28004771 10.1038/srep39223 PMC5177886

[95] Mund A, Coscia F, Kriston A, Hollandi R, Kovacs F, 2022. Deep visual proteomics defines single-cell identity and heterogeneity. Nat. Biotechnol 40:1231–40 35590073 10.1038/s41587-022-01302-5 PMC9371970

[96] Zhu Y, Clair G, Chrisler WB, Shen Y, Zhao R, 2018. Proteomic analysis of single mammalian cells enabled by microfluidic nanodroplet sample preparation and ultrasensitive NanoLC-MS. Angew. Chem. Int. Ed. Engl 57:12370–74 29797682 10.1002/anie.201802843 PMC6261339

[97] Dou M, Clair G, Tsai CF, Xu K, Chrisler WB, 2019. High-throughput single cell proteomics enabled by multiplex isobaric labeling in a nanodroplet sample preparation platform. Anal. Chem 91:13119–27 31509397 10.1021/acs.analchem.9b03349 PMC7192326

[98] Cong Y, Motamedchaboki K, Misal SA, Liang Y, Guise AJ, 2020. Ultrasensitive single-cell proteomics workflow identifies >1000 protein groups per mammalian cell. Chem. Sci 12:1001–6 34163866 10.1039/d0sc03636f PMC8178986

[99] Brunner AD, Thielert M, Vasilopoulou C, Ammar C, Coscia F, 2022. Ultra-high sensitivity mass spectrometry quantifies single-cell proteome changes upon perturbation. Mol. Syst. Biol 18:e10798 35226415 10.15252/msb.202110798 PMC8884154

[100] Specht H, Emmott E, Petelski AA, Huffman RG, Perlman DH, 2021. Single-cell proteomic and transcriptomic analysis of macrophage heterogeneity using SCoPE2. Genome Biol. 22:50 33504367 10.1186/s13059-021-02267-5 PMC7839219

[101] Pensold D, Zimmer-Bensch G. 2020. Methods for single-cell isolation and preparation. Adv. Exp. Med. Biol 1255:7–27 32949387 10.1007/978-981-15-4494-1_2

[102] LaBelle CA, Massaro A, Cortes-Llanos B, Sims CE, Allbritton NL. 2021. Image-based live cell sorting. Trends Biotechnol. 39:613–23 33190968 10.1016/j.tibtech.2020.10.006 PMC8113340

[103] Ibrahim SF, van den Engh G. 2007. Flow cytometry and cell sorting. Adv. Biochem. Eng. Biotechnol 106:19–39 17728993 10.1007/10_2007_073

[104] Emmert-Buck MR, Bonner RF, Smith PD, Chuaqui RF, Zhuang Z, 1996. Laser capture microdissection. Science 274:998–1001 8875945 10.1126/science.274.5289.998

[105] Espina V, Wulfkuhle JD, Calvert VS, VanMeter A, Zhou W, 2006. Laser-capture microdissection. Nat. Protoc 1:586–603 17406286 10.1038/nprot.2006.85

[106] Zhu Y, Dou M, Piehowski PD, Liang Y, Wang F, 2018. Spatially resolved proteome mapping of laser capture microdissected tissue with automated sample transfer to nanodroplets. Mol. Cell. Proteom 17:1864–74 10.1074/mcp.TIR118.000686 PMC6126383 29941660

[107] Ctortecka C, Hartlmayr D, Seth A, Mendjan S, Tourniaire G, Mechtler K. 2022. An automated workflow for multiplexed single-cell proteomics sample preparation at unprecedented sensitivity. bioRxiv 2021.04.14.439828. 10.1101/2021.04.14.439828 PMC10684380 37839701

[108] Leduc A, Huffman RG, Cantlon J, Khan S, Slavov N. 2022. Exploring functional protein covariation across single cells using nPOP. Genome Biol. 23:261 36527135 10.1186/s13059-022-02817-5 PMC9756690

[109] Zhu Y, Piehowski PD, Zhao R, Chen J, Shen Y, 2018. Nanodroplet processing platform for deep and quantitative proteome profiling of 10–100 mammalian cells. Nat. Commun 9:882 29491378 10.1038/s41467-018-03367-w PMC5830451

[110] Li ZY, Huang M, Wang XK, Zhu Y, Li JS, 2018. Nanoliter-scale oil-air-droplet chip-based single cell proteomic analysis. Anal. Chem 90:5430–38 29551058 10.1021/acs.analchem.8b00661

[111] Gebreyesus ST, Siyal AA, Kitata RB, Chen ES, Enkhbayar B, 2022. Streamlined single-cell proteomics by an integrated microfluidic chip and data-independent acquisition mass spectrometry. Nat. Commun 13:37 35013269 10.1038/s41467-021-27778-4 PMC8748772

[112] Lei Y, Tang R, Xu J, Wang W, Zhang B, 2021. Applications of single-cell sequencing in cancer research: progress and perspectives. J. Hematol. Oncol 14:91 34108022 10.1186/s13045-021-01105-2 PMC8190846

[113] Budnik B, Levy E, Harmange G, Slavov N. 2018. SCoPE-MS: mass spectrometry of single mammalian cells quantifies proteome heterogeneity during cell differentiation. Genome Biol. 19:161 30343672 10.1186/s13059-018-1547-5 PMC6196420

[114] Cheung TK, Lee CY, Bayer FP, McCoy A, Kuster B, Rose CM. 2021. Defining the carrier proteome limit for single-cell proteomics. Nat. Methods 18:76–83 33288958 10.1038/s41592-020-01002-5

[115] Huffman RG, Chen A, Specht H, Slavov N. 2019. DO-MS: data-driven optimization of mass spectrometry methods. J. Proteome Res 18:2493–500 31081635 10.1021/acs.jproteome.9b00039 PMC6737531

[116] Tsai CF, Zhao R, Williams SM, Moore RJ, Schultz K, 2020. An improved boosting to amplify signal with isobaric labeling (iBASIL) strategy for precise quantitative single-cell proteomics. Mol. Cell. Proteom 19:828–38 10.1074/mcp.RA119.001857 PMC7196584 32127492

[117] Williams SM, Liyu AV, Tsai CF, Moore RJ, Orton DJ, 2020. Automated coupling of nanodroplet sample preparation with liquid chromatography-mass spectrometry for high-throughput single-cell proteomics. Anal. Chem 92:10588–96 32639140 10.1021/acs.analchem.0c01551 PMC7793572

[118] Webber KGI, Truong T, Johnston SM, Zapata SE, Liang Y, 2022. Label-free profiling of up to 200 single-cell proteomes per day using a dual-column nanoflow liquid chromatography platform. Anal. Chem 94:6017–25 35385261 10.1021/acs.analchem.2c00646 PMC9356711

[119] Demichev V, Messner CB, Vernardis SI, Lilley KS, Ralser M. 2020. DIA-NN: neural networks and interference correction enable deep proteome coverage in high throughput. Nat. Methods 17:41–44 31768060 10.1038/s41592-019-0638-x PMC6949130

[120] Tsou CC, Avtonomov D, Larsen B, Tucholska M, Choi H, 2015. DIA-Umpire: comprehensive computational framework for data-independent acquisition proteomics. Nat. Methods 12:258–64 25599550 10.1038/nmeth.3255 PMC4399776

[121] Demichev V, Szyrwiel L, Yu F, Teo GC, Rosenberger G, 2022. dia-PASEF data analysis using FragPipe and DIA-NN for deep proteomics of low sample amounts. Nat. Commun 13:3944 35803928 10.1038/s41467-022-31492-0 PMC9270362

[122] Saha-Shah A, Esmaeili M, Sidoli S, Hwang H, Yang J, 2019. Single cell proteomics by data-independent acquisition to study embryonic asymmetry in *Xenopus laevis*. Anal. Chem 91:8891–99 31194517 10.1021/acs.analchem.9b00327 PMC6688503

[123] Stejskal K, Op de Beeck J, Durnberger G, Jacobs P, Mechtler K. 2021. Ultrasensitive nanoLC-MS of subnanogram protein samples using second generation micropillar array LC technology with Orbitrap Exploris 480 and FAIMS PRO. Anal. Chem 93:8704–10 34137250 10.1021/acs.analchem.1c00990 PMC8253486

[124] Meier F, Brunner AD, Frank M, Ha A, Bludau I, 2020. diaPASEF: parallel accumulation-serial fragmentation combined with data-independent acquisition. Nat. Methods 17:1229–36 33257825 10.1038/s41592-020-00998-0

[125] Woo J, Clair GC, Williams SM, Feng S, Tsai CF, 2022. Three-dimensional feature matching improves coverage for single-cell proteomics based on ion mobility filtering. Cell Syst. 13:426–34.e4 35298923 10.1016/j.cels.2022.02.003 PMC9119937

[126] Tsai CF, Wang YT, Hsu CC, Kitata RB, Chu RK, 2023. A streamlined tandem tip-based workflow for sensitive nanoscale phosphoproteomics. Commun. Biol 6:70 36653408 10.1038/s42003-022-04400-x PMC9849344

[127] Yi L, Tsai CF, Dirice E, Swensen AC, Chen J, 2019. Boosting to amplify signal with isobaric labeling (BASIL) strategy for comprehensive quantitative phosphoproteomic characterization of small populations of cells. Anal. Chem 91:5794–801 30843680 10.1021/acs.analchem.9b00024 PMC6596310

[128] Fulcher JM, Markillie LM, Mitchell HD, Williams SM, Engbrecht KM, 2022. Parallel measurement of transcriptomes and proteomes from same single cells using nanodroplet splitting. bioRxiv 2022.05.17.492137. 10.1101/2022.05.17.492137 PMC11621338 39638780

[129] Srinivasan S, Kalinava N, Aldana R, Li Z, van Hagen S, 2021. Misannotated multi-nucleotide variants in public cancer genomics datasets lead to inaccurate mutation calls with significant implications. Cancer Res. 81:282–88 33115802 10.1158/0008-5472.CAN-20-2151

[130] Koboldt DC. 2020. Best practices for variant calling in clinical sequencing. Genome Med. 12:91 33106175 10.1186/s13073-020-00791-w PMC7586657

[131] Zhao Y, Li MC, Konate MM, Chen L, Das B, 2021. TPM, FPKM, or normalized counts? A comparative study of quantification measures for the analysis of RNA-seq data from the NCI patient-derived models repository. J. Transl. Med 19:269 34158060 10.1186/s12967-021-02936-w PMC8220791

[132] Calderon-Celis F, Encinar JR, Sanz-Medel A. 2018. Standardization approaches in absolute quantitative proteomics with mass spectrometry. Mass Spectrom. Rev 37:715–37 28758227 10.1002/mas.21542

[133] Gay CM, Stewart CA, Park EM, Diao L, Groves SM, 2021. Patterns of transcription factor programs and immune pathway activation define four major subtypes of SCLC with distinct therapeutic vulnerabilities. Cancer Cell 39:346–60.e7 33482121 10.1016/j.ccell.2020.12.014 PMC8143037

[134] Zeng Z, Vo AH, Mao C, Clare SE, Khan SA, Luo Y. 2019. Cancer classification and pathway discovery using non-negative matrix factorization. J. Biomed. Inform 96:103247 31271844 10.1016/j.jbi.2019.103247 PMC6697569

[135] Hamamoto R, Takasawa K, Machino H, Kobayashi K, Takahashi S, 2022. Application of non-negative matrix factorization in oncology: one approach for establishing precision medicine. Brief. Bioinform 23:bbac246 10.1093/bib/bbac246 PMC9294421 35788277

[136] Masica DL, Karchin R. 2011. Correlation of somatic mutation and expression identifies genes important in human glioblastoma progression and survival. Cancer Res. 71:4550–61 21555372 10.1158/0008-5472.CAN-11-0180 PMC3129415

[137] Yang M, Petralia F, Li Z, Li H, Ma W, 2020. Community assessment of the predictability of cancer protein and phosphoprotein levels from genomics and transcriptomics. Cell Syst. 11:186–95.e9 32710834 10.1016/j.cels.2020.06.013

[138] Payne SH. 2015. The utility of protein and mRNA correlation. Trends Biochem. Sci 40:1–3 25467744 10.1016/j.tibs.2014.10.010 PMC4776753

[139] Arshad OA, Danna V, Petyuk VA, Piehowski PD, Liu T, 2019. An integrative analysis of tumor proteomic and phosphoproteomic profiles to examine the relationships between kinase activity and phosphorylation. Mol. Cell. Proteom 18:S26–36 10.1074/mcp.RA119.001540 PMC6692771 31227600

[140] Delgado FM, Gomez-Vela F. 2019. Computational methods for gene regulatory networks reconstruction and analysis: a review. Artif. Intell. Med 95:133–45 30420244 10.1016/j.artmed.2018.10.006

[141] Hernaez M, Blatti C, Gevaert O. 2020. Comparison of single and module-based methods for modeling gene regulatory networks. Bioinformatics 36:558–67 31287491 10.1093/bioinformatics/btz549

[142] Munk S, Refsgaard JC, Olsen JV, Jensen LJ. 2016. From phosphosites to kinases. Methods Mol. Biol 1355:307–21 26584935 10.1007/978-1-4939-3049-4_21

[143] Munk S, Refsgaard JC, Olsen JV. 2016. Systems analysis for interpretation of phosphoproteomics data. Methods Mol. Biol 1355:341–60 26584937 10.1007/978-1-4939-3049-4_23

[144] Liberzon A, Birger C, Thorvaldsdottir H, Ghandi M, Mesirov JP, Tamayo P. 2015. The Molecular Signatures Database (MSigDB) hallmark gene set collection. Cell Syst. 1:417–25 26771021 10.1016/j.cels.2015.12.004 PMC4707969

[145] Pauli C, Hopkins BD, Prandi D, Shaw R, Fedrizzi T, 2017. Personalized in vitro and in vivo cancer models to guide precision medicine. Cancer Discov. 7:462–77 28331002 10.1158/2159-8290.CD-16-1154 PMC5413423

[146] Beltran H, Eng K, Mosquera JM, Sigaras A, Romanel A, 2015. Whole-exome sequencing of metastatic cancer and biomarkers of treatment response. JAMA Oncol. 1:466–74 26181256 10.1001/jamaoncol.2015.1313 PMC4505739

[147] Lehmann S, Brede C, Lescuyer P, Cocho JA, Vialaret J, 2017. Clinical mass spectrometry proteomics (cMSP) for medical laboratory: What does the future hold? Clin. Chim. Acta 467:51–58 27265523 10.1016/j.cca.2016.06.001

[148] Rudnick PA, Markey SP, Roth J, Mirokhin Y, Yan X, 2016. A description of the Clinical Proteomic Tumor Analysis Consortium (CPTAC) common data analysis pipeline. J. Proteome Res 15:1023–32 26860878 10.1021/acs.jproteome.5b01091 PMC5117628

[149] Paik YK, Jeong SK, Omenn GS, Uhlen M, Hanash S, 2012. The Chromosome-Centric Human Proteome Project for cataloging proteins encoded in the genome. Nat. Biotechnol 30:221–23 22398612 10.1038/nbt.2152

[150] Brenes A, Hukelmann J, Bensaddek D, Lamond AI. 2019. Multibatch TMT reveals false positives, batch effects and missing values. Mol. Cell. Proteom 18:1967–80 10.1074/mcp.RA119.001472 PMC6773557 31332098

[151] Plubell DL, Kall L, Webb-Robertson BJ, Bramer LM, Ives A, 2022. Putting humpty dumpty back together again: What does protein quantification mean in bottom-up proteomics? J. Proteome Res 21:891–98 35220718 10.1021/acs.jproteome.1c00894 PMC8976764

[152] Webb-Robertson BJ, Wiberg HK, Matzke MM, Brown JN, Wang J, 2015. Review, evaluation, and discussion of the challenges of missing value imputation for mass spectrometry-based label-free global proteomics. J. Proteome Res 14:1993–2001 25855118 10.1021/pr501138h PMC4776766

[153] Monroe ME, Shaw JL, Daly DS, Adkins JN, Smith RD. 2008. MASIC: a software program for fast quantitation and flexible visualization of chromatographic profiles from detected LC-MS(/MS) features. Comput. Biol. Chem 32:215–17 18440872 10.1016/j.compbiolchem.2008.02.006 PMC2487672

[154] Kim S, Pevzner PA. 2014. MS-GFC makes progress towards a universal database search tool for proteomics. Nat. Commun 5:5277 25358478 10.1038/ncomms6277 PMC5036525

[155] Wright JC, Collins MO, Yu L, Kall L, Brosch M, Choudhary JS. 2012. Enhanced peptide identification by electron transfer dissociation using an improved Mascot Percolator. Mol. Cell. Proteom 11:478–91 10.1074/mcp.O111.014522 PMC3412976 22493177

[156] Wang M, Beckmann ND, Roussos P, Wang E, Zhou X, 2018. The Mount Sinai cohort of large-scale genomic, transcriptomic and proteomic data in Alzheimer’s disease. Sci. Data 5:180185 30204156 10.1038/sdata.2018.185 PMC6132187

[157] Tyanova S, Temu T, Sinitcyn P, Carlson A, Hein MY, 2016. The Perseus computational platform for comprehensive analysis of (prote)omics data. Nat. Methods 13:731–40 27348712 10.1038/nmeth.3901

[158] Clough T, Thaminy S, Ragg S, Aebersold R, Vitek O. 2012. Statistical protein quantification and significance analysis in label-free LC-MS experiments with complex designs. BMC Bioinform. 13(Suppl. 16):S6 10.1186/1471-2105-13-S16-S6 PMC3489535 23176351

[159] Stacklies W, Redestig H, Scholz M, Walther D, Selbig J. 2007. pcaMethods—a bioconductor package providing PCA methods for incomplete data. Bioinformatics 23:1164–67 17344241 10.1093/bioinformatics/btm069

[160] Perez-Riverol Y, Bai J, Bandla C, Garcia-Seisdedos D, Hewapathirana S, 2022. The PRIDE database resources in 2022: a hub for mass spectrometry-based proteomics evidences. Nucleic Acids Res. 50:D543–52 34723319 10.1093/nar/gkab1038 PMC8728295

[161] Jarnuczak AF, Vizcaino JA. 2017. Using the PRIDE database and ProteomeXchange for submitting and accessing public proteomics datasets. Curr. Protoc. Bioinform 59:13.31. 1–12 10.1002/cpbi.30 28902400

[162] Crusoe MR, Abeln S, Iosup A, Amstutz P, Chilton J, 2022. Methods included: standardizing computational reuse and portability with the Common Workflow Language. Commun. ACM 65:54–63

[163] Di Tommaso P, Chatzou M, Floden EW, Barja PP, Palumbo E, Notredame C. 2017. Nextflow enables reproducible computational workflows. Nat. Biotechnol 35:316–19 28398311 10.1038/nbt.3820

[164] Li C, Gao M, Yang W, Zhong C, Yu R. 2021. Diamond: a multi-modal DIA mass spectrometry data processing pipeline. Bioinformatics 37:265–67 33416868 10.1093/bioinformatics/btaa1093

[165] Walzer M, Garcia-Seisdedos D, Prakash A, Brack P, Crowther P, 2022. Implementing the reuse of public DIA proteomics datasets: from the PRIDE database to Expression Atlas. Sci. Data 9:335 35701420 10.1038/s41597-022-01380-9 PMC9197839

[166] Palmblad M, Lamprecht AL, Ison J, Schwammle V. 2019. Automated workflow composition in mass spectrometry-based proteomics. Bioinformatics 35:656–64 30060113 10.1093/bioinformatics/bty646 PMC6378944

[167] Latonen L, Afyounian E, Jylha A, Nattinen J, Aapola U, 2018. Integrative proteomics in prostate cancer uncovers robustness against genomic and transcriptomic aberrations during disease progression. Nat. Commun 9:1176 29563510 10.1038/s41467-018-03573-6 PMC5862881

[168] Altelaar AF, Heck AJ. 2012. Trends in ultrasensitive proteomics. Curr. Opin. Chem. Biol 16:206–13 22226769 10.1016/j.cbpa.2011.12.011

[169] Kelly RT. 2020. Single-cell proteomics: progress and prospects. Mol. Cell. Proteom 19:1739–48 10.1074/mcp.R120.002234 PMC7664119 32847821

[170] Slavov N 2022. Scaling up single-cell proteomics. Mol. Cell. Proteom 21:100179 10.1016/j.mcpro.2021.100179 PMC8683604 34808355

[171] Duncan KD, Fyrestam J, Lanekoff I. 2019. Advances in mass spectrometry based single-cell metabolomics. Analyst 144:782–93 30426983 10.1039/c8an01581c

[172] Lanekoff I, Sharma VV, Marques C. 2022. Single-cell metabolomics: Where are we and where are we going? Curr. Opin. Biotechnol 75:102693 35151979 10.1016/j.copbio.2022.102693

[173] Hu R, Li Y, Yang Y, Liu M. 2023. Mass spectrometry-based strategies for single-cell metabolomics. Mass Spectrom. Rev 42:67–94 34028064 10.1002/mas.21704

[174] Wu Z, Shen Y, Zhang X. 2022. TAG-TMTpro, a hyperplexing quantitative approach for high-throughput proteomic studies. Anal. Chem 94:12565–69 36066113 10.1021/acs.analchem.2c02099

[175] Dephoure N, Gygi SP. 2012. Hyperplexing: a method for higher-order multiplexed quantitative proteomics provides a map of the dynamic response to rapamycin in yeast. Sci. Signal 5:rs2 10.1126/scisignal.2002548 PMC5292868 22457332

[176] Welle KA, Zhang T, Hryhorenko JR, Shen S, Qu J, Ghaemmaghami S. 2016. Time-resolved analysis of proteome dynamics by tandem mass tags and stable isotope labeling in cell culture (TMT-SILAC) hyperplexing. Mol. Cell. Proteom 15:3551–63 10.1074/mcp.M116.063230 PMC5141271 27765818

[177] Brandi J, Noberini R, Bonaldi T, Cecconi D. 2022. Advances in enrichment methods for mass spectrometry-based proteomics analysis of post-translational modifications. J. Chromatogr. A 1678:463352 35896048 10.1016/j.chroma.2022.463352

[178] Hermann J, Schurgers L, Jankowski V. 2022. Identification and characterization of post-translational modifications: clinical implications. Mol. Aspects Med 86:101066 35033366 10.1016/j.mam.2022.101066

[179] Zecha J, Bayer FP, Wiechmann S, Woortman J, Berner N, 2023. Decrypting drug actions and protein modifications by dose- and time-resolved proteomics. Science 380:93–101 36926954 10.1126/science.ade3925 PMC7615311

[180] Flores-Morales A, Bergmann TB, Lavallee C, Batth TS, Lin D, 2019. Proteogenomic characterization of patient-derived xenografts highlights the role of REST in neuroendocrine differentiation of castration-resistant prostate cancer. Clin. Cancer Res 25:595–608 30274982 10.1158/1078-0432.CCR-18-0729

[181] Sinha A, Huang V, Livingstone J, Wang J, Fox NS, 2019. The proteogenomic landscape of curable prostate cancer. Cancer Cell 35:414–27.e6 30889379 10.1016/j.ccell.2019.02.005 PMC6511374

